# AarF Domain Containing Kinase 3 (ADCK3) Mutant Cells Display Signs of Oxidative Stress, Defects in Mitochondrial Homeostasis and Lysosomal Accumulation

**DOI:** 10.1371/journal.pone.0148213

**Published:** 2016-02-11

**Authors:** Jason K. Cullen, Norazian Abdul Murad, Abrey Yeo, Matthew McKenzie, Micheal Ward, Kok Leong Chong, Nicole L. Schieber, Robert G. Parton, Yi Chieh Lim, Ernst Wolvetang, Ghassan J. Maghzal, Roland Stocker, Martin F. Lavin

**Affiliations:** 1 QIMR Berghofer Medical Research Institute, Brisbane, QLD, Australia; 2 Hudson Institute of Medical Research, Centre for Genetic Diseases, Melbourne, VIC, Australia; 3 Mater Medical Research Institute, Glycation and Diabetic Complications Group, Translational Research Institute, Brisbane, QLD, Australia; 4 Queensland University of Technology, ARC Centre of Excellence for Free Radical Chemistry and Biotechnology, Brisbane, QLD, Australia; 5 The University of Queensland, Institute for Molecular Bioscience and Centre for Microscopy and Microanalysis, St. Lucia, QLD, Australia; 6 The University of Queensland, Australian Institute for Bioengineering and Nanotechnology, Brisbane, Australia; 7 Victor Chang Cardiac Research Institute, Vascular Biology Division, Darlinghurst, Australia; 8 UKM Medical Molecular Biology Institute, Kuala Lumpur, Malaysia; 9 The University of Queensland Centre for Clinical Research, Brisbane, QLD, Australia; University of Tasmania, AUSTRALIA

## Abstract

Autosomal recessive ataxias are a clinically diverse group of syndromes that in some cases are caused by mutations in genes with roles in the DNA damage response, transcriptional regulation or mitochondrial function. One of these ataxias, known as Autosomal Recessive Cerebellar Ataxia Type-2 (ARCA-2, also known as SCAR9/COQ10D4; OMIM: #612016), arises due to mutations in the *ADCK3* gene. The product of this gene (ADCK3) is an atypical kinase that is thought to play a regulatory role in coenzyme Q_10_ (CoQ_10_) biosynthesis. Although much work has been performed on the *S*. *cerevisiae* orthologue of ADCK3, the cellular and biochemical role of its mammalian counterpart, and why mutations in this gene lead to human disease is poorly understood. Here, we demonstrate that ADCK3 localises to mitochondrial cristae and is targeted to this organelle via the presence of an N-terminal localisation signal. Consistent with a role in CoQ_10_ biosynthesis, ADCK3 deficiency decreased cellular CoQ_10_ content. In addition, endogenous ADCK3 was found to associate *in vitro* with recombinant Coq3, Coq5, Coq7 and Coq9, components of the CoQ_10_ biosynthetic machinery. Furthermore, cell lines derived from ARCA-2 patients display signs of oxidative stress, defects in mitochondrial homeostasis and increases in lysosomal content. Together, these data shed light on the possible molecular role of ADCK3 and provide insight into the cellular pathways affected in ARCA-2 patients.

## Introduction

Coenzyme Q (CoQ) is a lipophilic electron and proton carrier that plays important roles during both development and ageing [1–4]. It plays several important functions within cells and consists of a benzoquinone ring, synthesised from phenylalanine or tyrosine, and a polyisoprenyl side chain, the length of which (Q_n_) varies from organism to organism [5]. CoQ_10_ is the major species observed in humans, whilst CoQ_9_ and CoQ_6_ are found predominantly in *C*. *elegans* and *S*. *cerevisiae*, respectively. Although mitochondria are believed to be the principle site of its biosynthesis (at least in yeast), a number of individual enzymes associated with CoQ production have been detected in the endoplasmic reticulum, Golgi and peroxisomes of animal cells [6–8]. In addition to its major role as an electron and proton carrier in the electron transport chain within mitochondria, CoQ is known to function as an antioxidant, regulate the activity of uncoupling proteins and modulate the physiochemical properties of membranes in which it resides [9, 10]. CoQ_10_ and its various analogs have also been shown to differentially modulate the activity of the mitochondrial permeability transition pore and may thus play a role in regulating apoptosis [11, 12].

The large majority of our knowledge on CoQ metabolism comes from studies in lower eukaryotes, typically budding yeast, where biosynthesis of CoQ_6_ is restricted to mitochondria and requires several genes (*COQ1-COQ9*). Interestingly, a plethora of genetic and biochemical evidence suggests that the products of these genes associate into a high molecular weight complex (~1MDa) within mitochondria [13–15]. Like the majority of mitochondrial proteins, the localisation of these species is dependent on the presence of an N-terminal mitochondrial targeting signal (MTS) [16]. Whilst the enzymatic function of Coq1, Coq2, Coq3, Coq5, Coq6 and Coq7 has been extensively studied and confirmed [17], the role Coq4, Coq8 and Coq9 play in CoQ_6_ biosynthesis is poorly understood. Human orthologues of these genes have been described and mutations in several of these are associated with the development of CoQ deficiency and disease. Indeed, mutations in *PDSS1*, *PDSS2*, *COQ2*, *COQ6* and *COQ9* are known to give rise to primary CoQ deficiency [18–23].

**Fig 1:**
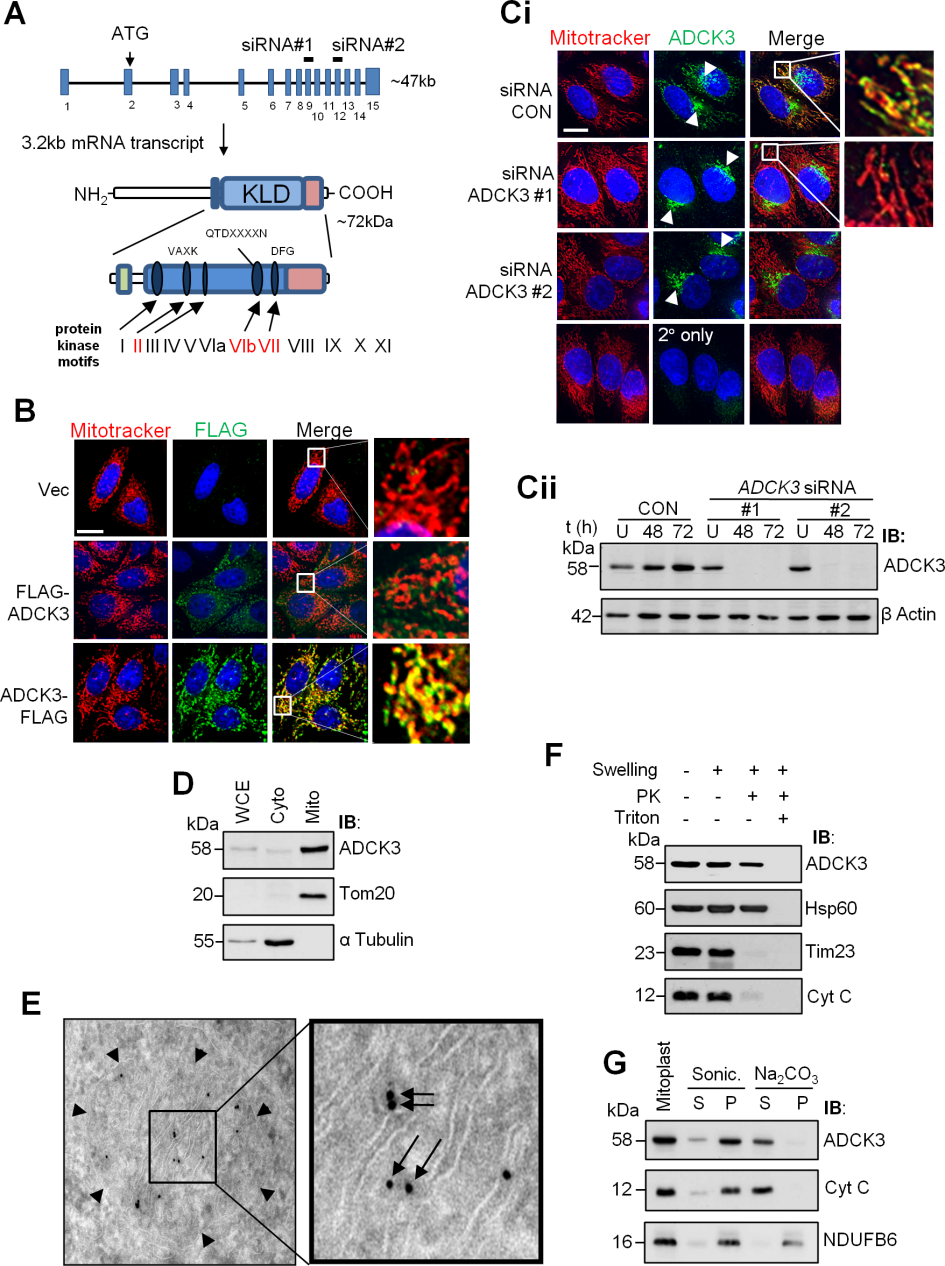
ADCK3 associates with mitochondrial cristae. **(A).** Schematic of the *ADCK3* gene and its product. The relative position of exons/introns within the *ADCK3* gene locus, the initiating ATG codon and the exons targeted by siRNA (*ADCK3* siRNA #1 and #2) are depicted. Isoform 1 of ADCK3 is also shown with the relative positions of several important domains and motifs. Green rectangle: Region conserved amongst ADCK family members and specifically related to CoQ biosynthesis. Blue rectangle: Kinase-Like Domain (KLD) which contains 5 of the 12 prototypical kinase motifs involved in ATP binding and the phospohtransfer reaction (Blue ovals). Red rectangle: C-terminal region, whilst highly conserved amongst the ADCK3/4 subgroup, is divergent from that found in classical protein kinases and other ADCK family members. **(B).** ADCK3-FLAG localises to mitochondria. HeLa cells transiently transfected with pcDNA3.1/Hygro(+) only (Vec) or pcDNA3.1/Hygro(+) containing *ADCK3* cDNA with either an N-terminal (pcDNA3.1/Hygro(+)-FLAG-*ADCK3*: FLAG-ADCK3) or C-terminal (pcDNA3.1/Hygro(+)-*ADCK3-*FLAG: ADCK3-FLAG) FLAG tag. Immunofluorescence performed with anti-FLAG and Alexa488-conjugated secondary antibodies (FLAG). Counterstaining for mitochondria was performed with Mitotracker Deep Red (Mitotracker). Nuclei were stained with Hoechst 3342. White bars: 15 μm. 63x mag. A single z-axis position is shown. See S1 Fig for images over multiple z-axis positions with ADCK3-EGFP. (**C, D).** Endogenous ADCK3 localises to mitochondria. Immunofluorescence (C) based analysis of HeLa cells conducted with anti-ADCK3 and Alexa488-conjugated secondary antibodies (ADCK3). HeLa cells treated with control siRNA (siRNA CON) or *ADCK3* siRNA #1 and #2 for 48 h. Again, counterstaining for mitochondria was performed with Mitotracker Deep Red (Mitotracker). Nuclei were stained with Hoechst 3342. White bar: 15 μm. Arrow head indicates the position of significant perinuclear or ‘golgi-like’ staining. 63x mag. Cellular subfractionation of HeLa cells (D) was also performed. WCE, cytoplasmic (Cyto) and mitochondrial (Mito) fractions (20 μg) were separated via SDS-PAGE and immunoblotting conducted with antibodies to ADCK3, Tom20 (a mitochondrial marker) and α Tubulin (a cytosolic marker). **(E).** ADCK3-FLAG localises to mitochondrial cristae. Anti-FLAG immunogold electron microscopy performed with HeLa cells transiently transfected with vector expressing ADCK3-FLAG. Arrow Heads: OMM; Arrows: ADCK3-FLAG on cristae. **(F, G).** Endogenous ADCK3 associates with the matrix side of the inner mitochondrial membrane. Proteinase K protection (E) and Sonication/Alkaline extraction experiments (F) were conducted with crude mitochondria. Immunoblotting of samples/fractions separated via SDS-PAGE was performed with antibodies to ADCK3, Hsp60 (matrix protein), Tim23 (integral IMM protein facing the IMS), Cyt C (IMM associated IMS protein) and NDUFB6 (integral IMM protein). PK: proteinase K. S: supernatant fraction. P: pellet fraction.

Recently, mutations in the human orthologue of *COQ8*, *ADCK3*, have reported to be associated with the development of primary CoQ_10_ deficiency and cerebellar ataxia [24–26]. Individuals with this condition, termed Autosomal Recessive Cerebellar Ataxia Type 2 (ARCA-2, also known as SCAR9 and COQ10D4. OMIM: #612016), exhibit childhood onset cerebellar atrophy with or without seizures, in addition to exercise intolerance. As expected, cell lines and muscle biopsies obtained from these patients display a reduced level of and capacity to synthesize CoQ_10_ [24–26]. Studies in yeast are consistent with these data. Indeed, *coq8* mutants have a decreased ability to form CoQ_6_ and accumulate the intermediate 3-hexaprenyl-4-hydroxybenzoic acid [27]. These data support a role for ADCK3 in CoQ_10_ biosynthesis, although the precise role it plays in this process and how defects give rise to ARCA-2 remain poorly understood. Interestingly, phylogenetic analysis suggests that ADCK3 belongs to a family of atypical kinases, with five members of the family present in humans (ADCK1-5) [24, 28]. *In silico* examination of ADCK3 primary structure shows that it contains five (I, II, III, VIb, VII) of 12 kinase motifs normally found in canonical protein kinases (Fig 1A and [24]). Given the presence of this ‘kinase-like’ domain, it has been speculated that ADCK3 plays a regulatory rather than catalytic role in CoQ_10_ biosynthesis. Recent data from *S*. *cerevisiae* support this notion. In fact, Coq8 has recently been shown to promote an interaction between Coq3 and the putative CoQ_6_ biosynthetic complex, with a number of Coq proteins being unphosphorylated in the absence of Coq8 [29, 30].

In this study we show that ADCK3 contains an N-terminal MTS and localises to the matrix side of mitochondrial cristae. Furthermore, we show that *adck3* mutant fibroblasts display increased sensitivity to H_2_O_2_, defects in mitochondrial homeostasis and possible OXPHOS complex remodelling, together with an increase in the lysosomal compartment. Together, these data shed light on ADCK3 function and highlight potential avenues to explore in determining the molecular basis of neurodegeneration in ARCA-2 patients.

## Materials and Methods

### Subcloning and site directed mutagenesis

The plasmids used in this study are detailed in Supporting Information (S1 Table). *ADCK3* cDNA was subcloned from pDNR-dual-*ADCK3* (Clone ID: HsCD00022398, plasmID database) into *EcoRI*/*BamHI* or *HindIII*/*BamHI* digested pcDNA3.1/Hygro(+) with either an N or C-terminal FLAG tag, producing pcDNA3.1/Hygro(+)-FLAG-*ADCK3* and pcDNA3.1/Hygro(+)-*ADCK3*-FLAG, respectively. To generate pEGFP-N3 based plasmids, full length cDNA or regions of *ADCK3* were amplified from pDNR-dual-*ADCK3* with the primers indicated in S2 Table. Resultant products were subcloned into pEGFP-N3 digested with *EcoRI*/*HindIII*. pGEM4Z-*ADCK3* was generated through insertion of full length ADCK3 cDNA into a BamHI site. pGEX-6P-1 derived plasmids for the expression of GST-tagged Coq3, Coq5, Coq7 and Coq9 were generated through cDNA amplification of the respective ORFs (including MTS regions) via PCR (see S1 and S2 Tables for primers and identity of template plasmids). PCR products were subsequently cloned into pGEX-6P-1 digested with either *EcoRI*/*BamHI* or *EcoRI*/*SalI*. Fidelity and identity of sequences in all constructs were confirmed by DNA sequencing.

### Cell culture, transfections and lentiviral transduction

HeLa and transformed control (C RSVt hTERT) and *adck3* mutant fibroblasts (P1 RSVt hTERT) were grown in DMEM (Life Science Technologies) supplemented with 12% foetal calf serum (FCS). All primary fibroblasts (previously detailed in [24]) were cultured in DMEM containing 15% FCS. Cells were maintained in a humidified incubator at 37°C, 5% CO_2_. All fibroblasts were used at a passage of p5 to p15 in all experiments. All media was supplemented with Pen/Strep. Treatment of cells with H_2_O_2_, ionizing radiation (IR), antimycin A and bafilomycin A1 (all from Sigma-Aldrich) was performed in complete media or Dulbecco's phosphate buffered saline (DPBS). Transfection of cells with constructs based on pcDNA3.1/Hygro(+) and pEGFP-N3 was performed using Lipofectamine 2000 reagent or Neon electroporation (Life Science Technologies) as per the manufacturer’s instructions. Lentiviral particles for the insertion of LC3-GFP into primary fibroblasts were produced as follows. Briefly, 293T cells were transfected with a combination of 2 packaging vectors and pEGFP-C1-LC3 using Lipofectamine^®^ 2000. After 24/48 h, media containing viral particles was collected, filtered (45 μm) and subsequently administered at various MOI to primary fibroblasts (60% confluency) cultured in T75 flasks.

### RNA interference

HeLa cells were transiently transfected with siRNA (Stealth siRNA, Life Science Technologies) targeted against ADCK3 (#1: 5’ CCUGAGAUUGUGGAUGAGCUCUGC 3’, #2: 5’ CCCAACUGGUCCAACUUCUUCUAUG 3’) or a scrambled control using Neon electroporation (Voltage: 1005 V, Pulse Width: 30 ms, Pulse #: 2) as per manufacturer’s instructions. siRNA duplexes were used at a final concentration of 10 mM during each knockdown experiment. After transfection, cells were maintained as described in media without Pen/Strep until harvest or analysis.

### Cell survival assays

The viability of *adck3* mutant fibroblasts (triplicate wells for each drug concentration) in response to IR/H_2_O_2_ treatment was determined by colony formation assay. Trypsinised cells were incubated with freshly prepared H_2_O_2_ for 30 min or irradiated with the indicated dose of IR before washing x2 with PBS and resuspending in culture medium. Cells were then cultured for 2–3 weeks to form colonies before staining with methylene blue and counting. Survival was expressed as the number of colonies in treated wells relative to colonies in untreated wells (% survival), as previously described [31].

### Analysis of coenzyme Q (CoQ) levels

Frozen cells (10^6^/500 μL PBS) were thawed and an aliquot (50 μL) of the resulting cell lysate was stored at -20°C until protein determination by the bicinchoninic acid assay. The remaining cell lysate (450 μL) was added to 1 mL ice-cold methanol and hexane (5 mL). The tube containing the original cell lysate was washed x3 with 100 μL PBS, with each wash being added to the methanol/hexane extraction mixture. The extract was mixed vigorously for 1 min (Vortex, maximal setting), and then centrifuged for 5 min at 1,500 x g and 4°C. The resulting hexane phase (4.5 mL) was removed and evaporated to dryness using a rotary evaporator, resuspended in 180 μL mobile phase, and 100 μL of the resulting organic extract were then subjected to liquid chromatography (HPLC) with electrochemical detection as described previously [32]. Ubiquinone-9, ubiquinol-9, ubiquinone-10 and ubiquinol-10 were quantified by their peak area comparison with authentic standards. The data obtained were standardized to either protein or non-esterified cholesterol, determined by HPLC-UV_214 nm_ of the organic extract as described previously [32].

### Immunoblotting and immunoprecipitation

Whole cell (WCEs) and mitochondrial extracts were prepared from cell/mitochondrial pellets using either RIPA buffer (50 mM Tris-HCl pH 7.4, 100 mM NaCl, 1% DOC, 1% SDS, 1% NP-40) or HBST (50 mM HEPES pH 7.4, 150 mM NaCl, 1% Triton-X100). Buffers were supplemented with 1x Complete Protease Inhibitor cocktail (Roche), 1 mM phenylmethanesulfonyl fluoride (PMSF), 200 mM β-glycerophosphate, 100 mM NaF and 1 mM sodium orthovanadate. Following incubation at 4°C for 1 h, insoluble material was removed via centrifugation and protein concentration determined using a DC assay kit (BioRad). Immunoprecipitations were performed with 1–2 mg of WCE. Briefly, WCE was pre-cleared with proteinA or G-agarose for 30 min. The precleared extract was incubated with antibody overnight at 4°C with agitation, after which immunoglobulin-protein complexes were captured with proteinA or G-coupled agarose for 2 h. After x3 washes in HBST, immunoprecipitates were used in further experimentation or analysed via SDS-PAGE/immunoblotting. The following antibodies were used in this study: rabbit anti-ADCK3 (Sigma-Aldrich; HPA018217; 1:1000), mouse monoclonal anti-FLAG M2 (Sigma-Aldrich: F3165; 1:1000); mouse anti-β actin (Sigma-Aldrich; A1978; 1:1000), mouse anti-Tom20 (BD Biosciences: 612278; 1:1000), mouse anti-Hsp60 (BD Biosciences: 611563; 1:1000) mouse anti-α tubulin (Sigma-Aldrich), mouse anti-Tim23 (BD Biosciences: 611222; 1:1000), mouse anti-NDFUB6 (a kind gift from Dr. M. McKenzie; 1:1000), sheep anti-Cyt C (Cell Signalling Technology; 4272; 1:1000), rabbit anti-LC3B (Cell Signalling Technology; 2775; 1:1000), mouse anti-GAPDH (Millipore; MAB374; 1:1000) and mouse anti-PCNA (Calbiochem; NA03; 1:1000).

### Immunofluorescence

Cells plated on coverslips/chamber slides/wells in preparation for immunofluorescence were first washed x3 with PBS. They were then fixed with 3% paraformaldehyde (PFA) in PBS pH 7.2 for 15 min prior to permeabilisation with 0.5% Triton-X, PBS pH 7.2 for 10 min and subsequent blocking with 2% BSA, 0.5% Triton-X, PBS pH 7.2 for 30 min. Incubations with primary antibody were performed in blocking buffer, typically at 1:100 dilution, overnight at 4°C. Alexa488 conjugated secondary antibodies were used at 1:750, again in blocking buffer for 1 h at 37°C. Coverslips were mounted onto glass slides using VectorShield medium with DAPI. MitoTracker^®^ Deep Red staining of HeLa and primary fibroblasts was performed in complete media using 200 nM of probe for 20 min. Lysosomal staining was performed with 200 nM LysoTracker^®^ Red for 15 min in complete media. Cells were subsequently washed x3 with PBS after which they were incubated in complete media for 30 min prior to fixation and visualisation. Images were acquired using a Deltavision Wide Field microscope and deconvoluted using Softworx software (Applied Precision).

### Subcellular fractionation and isolation of mitochondria

Subcellular fractionation was performed using a commercially available kit as previously described [33]. Briefly, 2x10^7^ HeLa cells were pelleted and resuspended in 800 μL Buffer A containing 1 mM PMSF. After vortexing for 5 sec at medium speed, the cell suspension was incubated on ice for 2 min to allow hypotonic swelling. 10 μL of Buffer B was then added and the suspension vortexed at maximum speed for 5 sec. The suspension was vortexed for 5 sec every min for a total of 5 min. 800 μL Buffer C was added to stabilise mitochondria and unbroken cells/nuclei removed through centrifugation for 10 min at 700 x g, 4°C. After another round of centrifugation using the above conditions, heavy mitochondria were isolated from the post nuclear supernatant, again by centrifugation for 15 min at 5,000 x g, 4°C. The supernatant from this step was the cytosolic fraction. The mitochondrial pellet was washed with 800 μL MSHE buffer (20 mM HEPES pH 7.2, 1 mM EDTA, 140 mM mannitol, 70 mM sucrose), centrifuged again at 12,000 x g for 5 min, 4°C and then resuspended in 100 μL MSHE buffer. Protein concentration in each fraction was determined via DC assay and equal amounts of protein from WCE, cytosolic and mitochondrial fractions precipitated using TCA. Protein pellets were resuspended in 1x SDS-loading buffer prior to separation by SDS-PAGE and immunoblotting. Mitochondria isolated via this method were also used as material for immunoprecipitations, and submitochondrial fractionation experiments.

### Proteinase K protection and sonication/alkaline extraction

Proteinase K protection experiments were performed using mitoplasts, generated from mitochondrial isolates as previously detailed [34]. 100 μg of mitoplasts, resuspended in 100 μL MSHE buffer, were incubated with 50 μg/mL proteinase K with or without 1% Triton-X100 for 30 min at room temperature. Proteinase K was inactivated on ice with 1 mM PMSF for 10 min prior to TCA precipitation. Untreated mitoplasts were used as a control. For sonication experiments, 500 μg of mitoplasts in 500 μL of MSHE were disrupted with a Branson Sonifier (Intensity 4, 30% duty cycle, 1 min). Sonicated membranes were pelleted by centrifugation at 100,000 x g and the supernatant fraction removed. Isolated membranes were resuspended in an identical volume of MSHE buffer and protein extracted from both fractions via TCA precipitation as above. Alkaline extraction was performed using 500 μg mitoplasts, resuspended in 500 μL 0.1 M Na_2_CO_3_ pH 11.5 and incubated on ice for 30 min. Supernatant and membrane fractions were subsequently acquired as described for sonication experiments. Equal amounts of protein were analysed by SDS-PAGE and immunoblotting performed as described above. Again, untreated mitoplasts were used as a control.

### Mitochondrial import assays

Mitochondria were first isolated from HeLa cells as previously detailed [35]. ^35^S labelled ADCK3 was translated *in vitro* using the TnT system (Promega), ^35^S-methionine and pGEM4Z containing *ADCK3* cDNA sequence. Equal amounts of radiolabelled protein were incubated with 100 μg purified mitochondria in the presence/absence of carbonyl cyanide 3-chlorophenylhydrazone (CCCP) for various times and at various temperatures. Following incubation, mitochondria were divided in two, pelleted via centrifugation at 12,000 x g, washed with MSHE and resuspended in MSHE with or without 10 mg/mL proteinase K. After incubation at room temperature for 10 min, proteinase K was inactivated on ice with 1 mM PMSF for a further 10 min prior precipitation of proteins with TCA. Pellets were resuspended in 1x SDS-loading buffer prior to separation of proteins by SDS-PAGE and autoradiography.

### N-terminal sequencing

Immunoprecipitated ADCK3-FLAG, isolated from HeLa cells transiently transfected with pcDNA3.1/Hygro(+)-*ADCK3*-FLAG, was separated using a modified SDS-PAGE procedure [36]. Following transfer to PVDF, the blot was stained with Coomassie Blue and the band corresponding to mature ADCK3-FLAG excised. N-terminal sequencing was subsequently performed by Edman degradation.

### Live cell imaging

HeLa cells transfected with pEGFP-N3 based constructs (see S1 Table) and plated in 8-well chamber slides (Ihbidi) were stained with MitoTracker^®^ Deep Red and Hoechst 3342 prior to visualisation using a Deltavision Wide Field microscope (Applied Precision). The stage was encapsulated by an environmental chamber set to 37°C and 5% CO_2_ was supplied to the cells during visualisation.

### Assesment of cytosolic and mitochondrial reactive oxygen and nitrogen species, mitochondrial mass, ΔΨm, lysosomal content and respiration rates

All primary cell cultures were grown to 80–90% confluency prior to experimentation. 24 h prior to analysis, fresh media was added to cells. For assessment of cytoplasmic and mitochondrial reactive oxygen and nitrogen species (ROS/RNS), 2x10^5^ cells were incubated in 500 μL DPBS containing either 10 μM CM-H_2_-DCFDA (5-(and-6)-chloromethyl-2',7'-dichlorodihydrofluorescein diacetate, acetyl ester) or 5 μM mitoSOX (Life Science Technologies) at 37°C, 5% CO_2_ for 30 or 15 min, respectively. Mitochondrial mass was determined by staining 2x10^5^ cells with 1 μM nonyl acridine orange in 500 μL PBS for 10 min. ΔΨm was analysed using JC-1 dye. Here, 2x10^5^ cells were incubated in 500 μL DPBS containing 5 μg/mL JC-1 for 15 min. Lysosomal content was analysed by incubating 2x10^5^ cells with either 100 nM LysoTracker^®^ Red in complete media or 1 μg/mL acridine orange in PBS. Stained cells were isolated via centrifugation at 335 x g for 4 min, resuspended in 1 mL of pre-warmed PBS and immediately analysed via flow cytometry using a BD FACsCalibur or Fortessa LSR flow cytometer (Becton Dickinson).

β-Galactosidase staining was performed using 1x10^4^ cells in 96-well plates. After 24h incubation at 37°C, 5% CO_2_, cells were partially fixed with 3.7% formaldehyde for 5 min, after which they were washed with PBS x 2 and then incubated overnight at 37°C in freshly prepared staining buffer (1 mg/mL X-gal, 5 mM K_3_Fe[CN]_6_, 5 mM K_4_Fe[CN]_6_, 2 mM MgCl_2_ in PBS pH 6.0 or citrate buffered saline pH 4.5). Following incubation cells were washed, again with PBS and images acquired via light microscopy.

Respiratory measurements (2x10^4^ cells per well from each cell line) were performed with an XF24 Extracellular Flux Analyzer from Seahorse Biosciences as per the manufacturer´s instructions. 1 μM of oligomycin, FCCP, rotenone and antimycin A were used in all experiments. Readings per well were normalised to cell number, as determined via the use of CyQUANT (Life Technologies).

### Blue Native (BN)-PAGE analysis of OXPHOS (super)complex stability

BN-PAGE was performed as described with minor modifications [37]. Mitochondria (50 μg protein) were solubilized for 30 min on ice in 50 μL of 20 mM Bis-Tris pH 7.4, 50 mM NaCl, 10% (v/v) glycerol containing either 1% (w/v) digitonin (Merck) or 1% (v/v) Triton X-100 (Sigma). Insoluble material was removed by centrifugation at 18,000 x g for 5 min at 4°C, with the soluble component combined with BN-PAGE loading dye (final concentrations: 0.5% (w/v) Coomassie Blue G, 50 mM ε-amino n-caproic acid (Sigma), 10 mM Bis-Tris pH 7.0) and separated on a 4–13% acrylamide-bisacrylamide BN-PAGE gel made up in 70 mM ε-amino n-caproic acid, 50 mM Bis-Tris (pH 7.0). For separation, cathode buffer (15 mM Bis-Tris pH 7.0, 50 mM tricine) containing 0.02% (w/v) Coomassie Blue G was used until the dye front had reached approximately one-third of the way through the gel before exchange with colourless cathode buffer. Anode buffer contained 50 mM Bis-Tris pH7.0. Native complexes were separated at 100 V / 5 mA for 13.5 h at 4°C. For two-dimensional PAGE, BN-PAGE strips were positioned into the stacker of a 10–16% polyacrylamide Tris-Tricine gradient gel [38, 39]. Samples were separated in the 2^nd^ dimension at 100 V / 25 mA for 14 h.

### Analysis of 4-hydroxy-nonenal (4-HNE) Michael Adducts and protein nitrotyroyslation (nitro-Y)

Primary fibroblasts were plated in 6-well plates containing pre-sterilised glass coverslips. Following 48 h incubation in complete media, cells were fixed with 4% paraformaldehyde and processed as described above in the immunofluorescence section using antibodies directed against 4-HNE Michael Adducts (1:100; Calbiochem) and nitrotyrosinylated protein (nitro-Y– 1:100; Cell Signaling Technology). An anti-rabbit Alexa568 antibody was used for fluorescence based imaging with a Zeiss AxioScope microscope under 40 x magnification. Fluorescence intensity values were acquired from 50 randomly selected cells using Image J and compared to control values. Three independent experiments were performed for each of the cell lines analysed.

### Electron Microscopy

HeLa cells transiently transfected with ADCK3-FLAG were fixed with 8% paraformaldehyde in phosphate buffer and processed for frozen sectioning according to published techniques [40]. Immunogold EM detection of the FLAG tag was performed on ultrathin frozen sections using anti-FLAG tag antibodies followed by 10 nm proteinA-gold. Primary fibroblasts were fixed in 2.5% glutaraldehyde in PBS and then processed for epon embedding. Sections were cut parallel to the culture substratum and post-stained on grid with uranyl acetate and lead citrate.

### Expression and isolation of GST-Coq fusion proteins

pGEX-6P-1-derived plasmids (S1 Table) were first transformed into *Escherichia coli* BL21 (DE3) cells (Lucigen). Transformed bacteria were cultured overnight at 37°C and subsequently used to seed expression cultures in 1 L 2xYT media. Expression of GST-Coq fusion proteins was induced at OD_595_ ~0.6–0.8 by the addition of 0.3 mM isopropyl β-D-1-thiogalactopyranoside (IPTG). After 3 h incubation at 37°C, cells were isolated via centrifugation, washed x1 with PBS pH 7.4 and resuspended in 10 mL of 1 x STE buffer (10 mM Tris-HCl pH 7.4, 100 mM NaCl, 1 mM EDTA) containing 1x Complete Protease Inhibitor and 1 mg/mL lysozyme IV. The cell suspension was incubated on ice for 30 min and then frozen at -80°C. Following this, 10 mL sonication/lysis buffer (1 x STE, 2 mM PMSF, 10 mM dithiothreitol (DTT)) was added, followed by the addition of 1.5% sarkosyl. After another freeze/thaw cycle, the cell suspension was sonicated, 2% Triton X-100 added and the bacterial lysate incubated at 4°C for 30 min. Insoluble material was removed via centrifugation and the supernatant incubated with 4 mL 50:50 slurry of glutathione agarose beads overnight at 4°C. Glutathione agarose bound GST fusion proteins were washed x3 with 1 x STE containing 1 mM PMSF and 5 mM DTT and stored at 4°C until use. GST fusion proteins for *in vitro* pull down experiments with mitochondrial extracts were left bound to glutathione agarose but equilibriated in HBST buffer with Complete Protease Inhibitor, 200 mM β-glycerophosphate and 1 mM sodium orthovanadate.

### *In vitro* pull down assays with GST-Coq substrates and mitochondrial extracts

Mitochondria from HeLa cells were isolated using a Miltenyi mitochondria isolation kit as described by the manufacturer (Miltenyi). Isolated mitochondria were solubilised in HBST containing complete protease inhibitor cocktail, 200 mM β-glycerophosphate and 1 mM sodium orthovanadate. Mitochondrial extract (1 mg) was subsequently added to glutathione agarose bead slurries containing ~2 μg of GST, GST-Coq3, GST-Coq5, GST-Coq7 or GST-Coq9. The resultant mixtures were incubated overnight at 4°C on a rotary wheel after which GST-Coq protein complexes were pelleted via centrifugation at 5,000 x g for 1 min. The pellets were washed x3 with HBST prior to analysis via SDS-PAGE and immunoblotting. Mitochondrial extract (10 μg) was analysed as a control.

### Statistical analysis

All data is displayed as mean ± standard error of the mean (S.E.M), unless otherwise indicated. Statistical analysis of all data was performed using either Prism® 6.0 software (GraphPad Software Inc.) or SPSS (IBM). n = 3 for all experiments unless otherwise indicated. Comparison subgroups in all respiratory measurement experiments (C vs. P group) were not normally distributed (Shapiro-Wilk normality test p-values < 0.05) hence a non-parametric test (Wilcoxon Mann Whitney test) was used in the analysis of this data (SPSS 1.2). All other data was analysed using the Student’s t-test (Prism® 6.0). Significance was defined as *p* < 0.05 in all cases.

## Results

### ADCK3 associates with mitochondrial cristae

A previous report had suggested that exogenously expressed ADCK3 localises to the mitochondrial compartment [41]. To confirm this, we transfected HeLa cells with ADCK3 containing a FLAG tag that had been fused to either the N or C-terminus of the protein (FLAG-ADCK3 and ADCK3-FLAG, respectively) and performed immunofluorescence with anti-FLAG antibody. Interestingly, ADCK3-FLAG colocalised with MitoTracker^®^ Deep Red but FLAG-ADCK3 did not, suggesting that ADCK3 may be processed at its N-terminus on, or prior to, import into mitochondria (Fig 1B). Almost complete colocalisation between an EGFP-tagged form of ADCK3 and MitoTracker^®^ Deep Red was also observed at multiple z-axis positions, suggesting that the large majority of exogenously expressed protein was indeed mitochondrial (S1A Fig). Use of an antibody directed against ADCK3 led to detection of a major band at ~58kDa in HeLa cell extracts that was no longer detected after treatment with ADCK3 RNAi (Fig 1Cii, S2A Fig). Furthermore, the apparent molecular weight of ~58kDa was lower than the 72kDa predicted for full length ADCK3 (Fig 1A), again suggestive of processing. This band was observed in a number of commonly used cells lines, including those of neuronal origin (S2B Fig). ADCK3-FLAG could also be immunoprecipitated from HeLa cell extracts with this antibody, further confirming its specificity (S2C Fig). We subsequently confirmed the mitochondrial localisation of endogenous ADCK3 by both immunofluorescence and subcellular fractionation (Fig 1Ci and 1D, respectively. Also see S1B Fig for multiple z-axis slices). We noticed that a large perinuclear or ‘Golgi-like’ staining pattern, similar to that described for Coq6 [23], was observed by immunofluorescence in HeLa cells using the ADCK3 antibody. This was shown, however, to be non-specific via the use of RNAi (Fig 1Ci). A similar profile was also observed in *adck3* mutant fibroblasts (Table 1: especially P1 cells, where protein is undetectable via immunoblotting; S3 Fig and see below). Interestingly, immunogold electron microscopy on frozen sections of HeLa cells transfected with ADCK3-FLAG showed localization of exogenously expressed protein to mitochondrial cristae (Fig 1E). We confirmed this for the endogenous protein using proteinase K protection experiments with mitoplasts, which clearly showed that ADCK3 was present in the mitochondrial matrix (Fig 1F). Moreover, the majority of ADCK3 remained associated with the inner membrane fraction after sonication but was present in the supernatant on alkaline extraction, suggesting that it was only peripherally associated with the inner mitochondrial membrane (IMM) (Fig 1G).

**Table 1:** *adck3* mutant cell lines used in this study.

Cell Line	*ADCK3* Mutation	Predicted αα change
P1	Homo c.1398+2T→C	D420WfsX40 or I467AfsX22
P2	Homo c.500_521 delinsTTG	Q167LfsX36 homozygous
P3	Hetero c.[1541A→G] + [1750_1752 delACC]	Y514C,T584 del

The cell lines used in this study have previously been described in [24].

**Fig 2:**
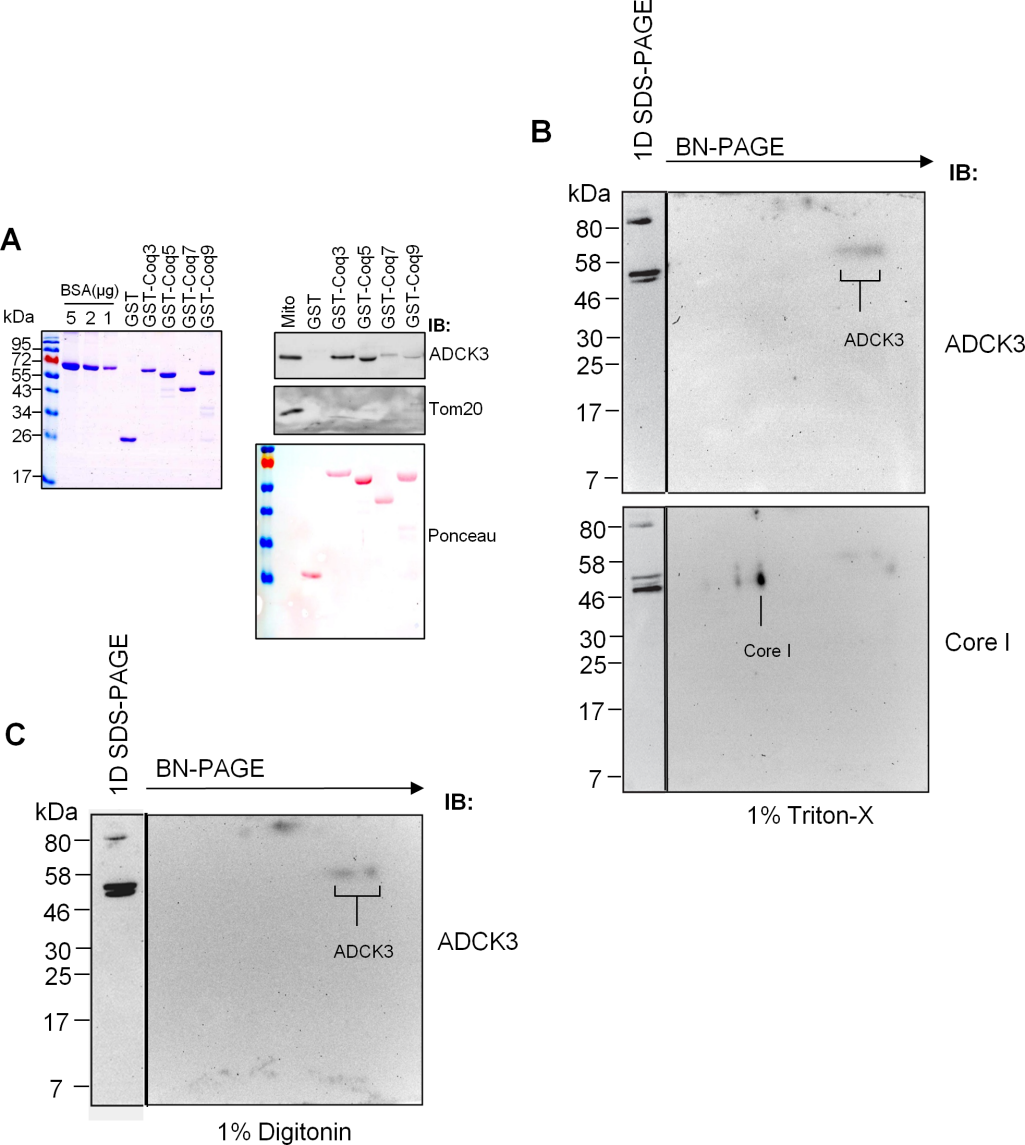
ADCK3 binds members of the CoQ biosynthetic complex *in vitro*. **(A).** ADCK3 interacts with Coq3, 5, 7 and 9 *in vitro*. Recombinant GST-Coq proteins were expressed and purified (left panel–Collodial Coomassie Staining) prior to use in pull down assays with mitochondrial extracts from HeLa cells (right panel). Ponceau staining was performed to identify GST-Coq proteins and their concentration. Immunoblotting was also performed with anti-ADCK3 and anti-Tom20 antibodies. **(B, C).** BN-PAGE data from Triton-X100 (B) and Digitonin (C) solubilised mitochondria from HEK293 cells. 50 μg mitochondria were solubilised and separated via 2D-BN-PAGE. Immunoblotting performed with antibodies to ADCK3 and Core I (Complex III component). 1D SDS-PAGE of mitochondrial extracts was performed as a control for antibody staining (see panels to left of 2D gels).

Given that Coq8, the yeast orthologue of ADCK3, has been shown to indirectly affect the phosphorylation state of Coq3, Coq5 and Coq7, we examined whether it could associate with the human orthologues of these proteins *in vitro*. Our pull down experiments with recombinant protein and mitochondrial extracts from HeLa cells suggested that ADCK3 interacted *in vitro* with GST-Coq3, GST-Coq5, GST-Coq7 and, interestingly, GST-Coq9 (Fig 2A). It did not associate with GST itself, however, suggesting that the observed interaction between ADCK3 and the various Coq proteins was specific. We also assessed whether ADCK3 resided in a high molecular weight complex using mitochondrial extracts from HEK293 cells and two dimensional (2D) BN-PAGE. Consistent with previous observations which suggested that Coq8 can homodimerise [29], we found that ADCK3 migrated as two low molecular weight spots under the native separation (at ~58KDa in the SDS-PAGE analysis of both Triton X-100 and digitonin solubilised mitochondrial extracts) that most probably represented monomeric and dimeric protein (Fig 2B and 2C). However, we failed to identify any pattern consistent with the association of endogenous protein in a high molecular weight structure. Together, these data demonstrated that ADCK3 is mitochondrial, is peripherally associated with the matrix side of cristae and interacts with components of the CoQ biosynthetic complex *in vitro*.

### ADCK3 contains an N-terminal MTS

**Fig 3:**
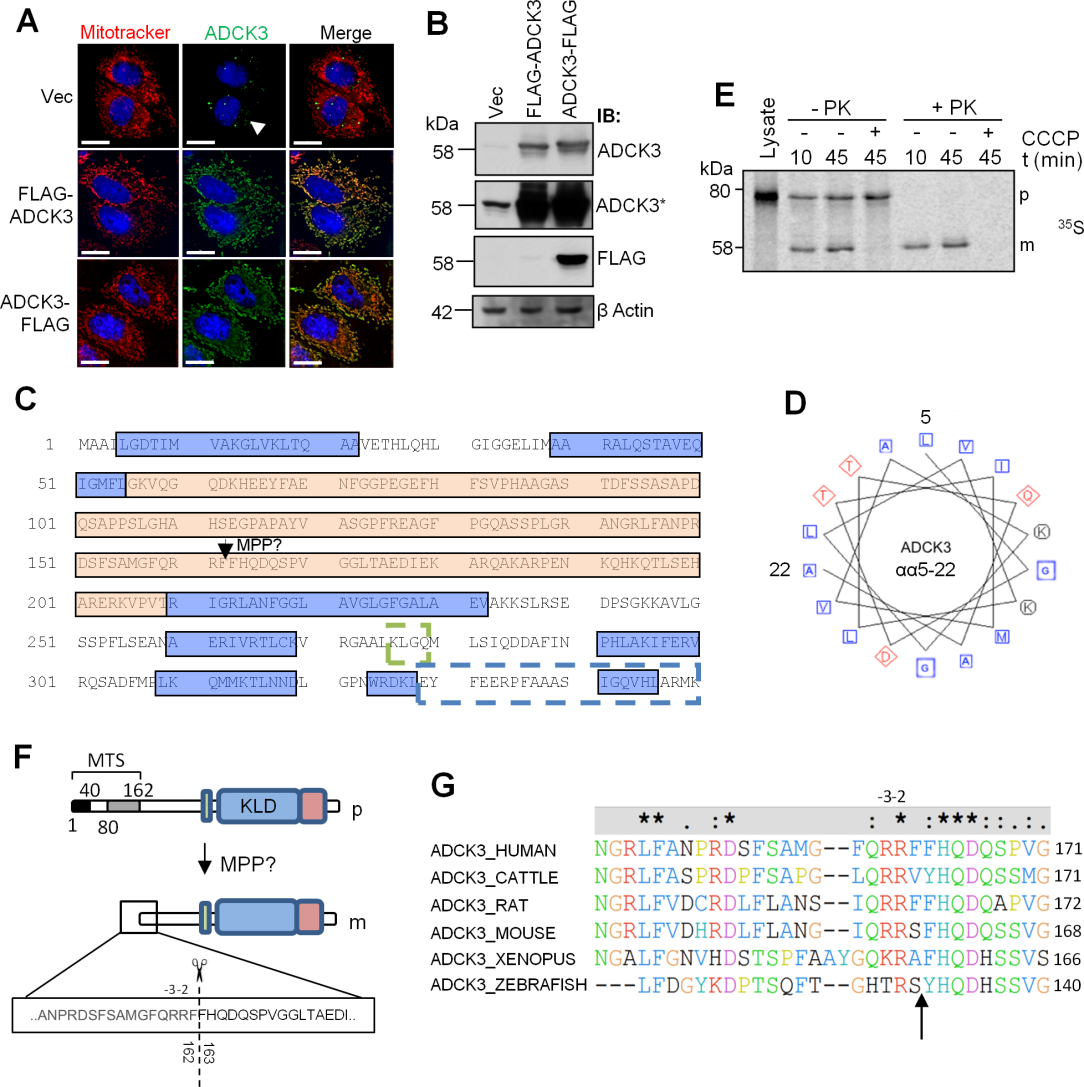
ADCK3 contains an N-terminal MTS. **(A, B).** An N-terminal FLAG tag does not prevent entry of ADCK3 into mitochondria. HeLa cells transiently transfected with pcDNA3.1/Hygro(+) only (Vec), pcDNA3.1/Hygro(+)-FLAG-*ADCK3* (FLAG-ADCK3) or pcDNA3.1/Hygro(+)-*ADCK3-*FLAG (ADCK3-FLAG). Immunofluorescence (A) or immunoblotting of WCEs (B) was performed with antibodies to ADCK3, FLAG and β actin. ADCK3* panel is a longer time exposure. Counterstaining for mitochondria was performed with Mitotracker Deep Red (Mitotracker). Nuclei were stained with Hoechst 3342. White bars: 15 μm. Note: The absence of peri-nuclear staining in Vec transfected cells was due to the reduced exposure time needed to prevent saturation of fluorescence from FLAG-ADCK3 and ADCK3-FLAG transfected cells. **(C).** ADCK3 contains two predicted alpha helical domains at its N-terminus. ADCK3 primary sequence (only αα1–350 are shown) was inserted into the online secondary structure prediction tools PROF and PNSS (ProteinPredict server). Blue boxes: predicted alpha helical regions. Orange box: Region of non-regular secondary structure. Dashed green box: Motif specifically related to CoQ biosynthesis. Dashed blue box: kinase-like domain. Arrow indicates the position of a possible MPP site. **(D).** A helical wheel arrangement of the N-terminal region of ADCK3 shows slight amphipathicity. The amino acids from the first predicted alpha helix described in C were inserted into an online tool to produce the helical wheel arrangement of residues depicted (see Experimental Procedures). Blue Squares: nonpolar residues. Red Diamonds: acidic residues. Black circles: basic residues. **(E).** ADCK3 undergoes a single cleavage event on entry into mitochondria. *In vitro* import of S^35^-methionine labelled ADCK3 into isolated mitochondria. 10 μM CCCP used to ablate ΔΨm. Lysate lane shows separation of *in vitro* translated protein only. Autorad image is depicted. p: precursor. m: mature protein. PK: proteinase K. **(F).** N-terminal sequencing of mature ADCK3. Schematic of ADCK3 indicating the position of the cleavage event observed in B as determined by N-terminal sequencing. R residues observed at positions -2, -3 and -13 are consistent with the known features of an MPP cleavage site. **(G).** Alignment of cleavage sequence positions amongst ADCK3 orthologues.

The above experiments (Fig 1B) suggested that the N-terminus of ADCK3 was processed on mitochondrial import. To ensure that the N-terminal FLAG tag was not restricting entry of ADCK3 into mitochondria, we transfected HeLa cells with FLAG-ADCK3 or ADCK3-FLAG and performed immunofluorescence and immunoblotting using the anti-ADCK3 antibody. As expected, ADCK3 colocalised with MitoTracker^®^ Deep Red staining in all samples, except in the vector only control (Fig 3A). Furthermore, immunoblot analysis confirmed the presence of processed ADCK3 but absence of the N-terminal FLAG tag after overexpression of FLAG-ADCK3 (Fig 3B). Together, these data suggest that the N-terminus of ADCK3 was processed on entry into mitochondria and that the N-terminal tag did not restrict import of exogenously expressed protein. *In silico* analysis of ADCK3 primary sequence using an online secondary structure prediction tool (PredictProtein server [42]—PROFphd) subsequently identified two possible alpha helical domains from amino acids 4–22 and 39–46, followed by a region of non-regular secondary structure (Fig 3C). A helical wheel arrangement of 15 amino acids from the first of these predicted alpha helices also showed slight amphipathicity (Fig 3D). Thus, the N-terminal region of ADCK3 displayed features consistent with known MTS regions [16].

To confirm the above analysis and to investigate whether ADCK3 underwent multiple processing steps on import, we performed an *in vitro* import experiment with isolated mitochondria and [^35^S]-methionine-labelled ADCK3. Radiolabelled precursor (p) was rapidly imported into energized mitochondria, resulting in the formation of a proteinase K insensitive, mature ADCK3 protein band (m) as quickly as 10min post addition (Fig 3E). Mature protein migrated at ~58kDa size, consistent with immunoblotting data of whole cell or mitochondrial extracts. The precursor migrated to a position much higher than the predicted size of full length ADCK3, *i*.*e*. ~80KDa, as observed in our earlier experiments (S2C Fig). As expected, import and processing of ADCK3 was prevented by dissipating mitochondrial membrane potential (ΔΨ_m_) with CCCP. No intermediate was observed during import, even after performing the experiment at lower temperatures (S4 Fig). N-terminal sequencing of mature protein (ADCK3-FLAG) isolated from whole cell extracts using anti-FLAG antibody subsequently revealed that ADCK3 was cleaved between two phenylalanine residues at amino acids 162 and 163 to produce mature protein (Fig 3F). The sequence showed strong similarity to the consensus sequence of the mitochondrial processing peptidase, MPP, *i*.*e*. an R residue at positions -2/3 [43] and was highly conserved throughout evolution (Fig 3G). Thus, ADCK3 contained a relatively large N-terminal MTS that was cleaved once at a possible MPP site during import into mitochondria.

To further probe the function of the ADCK3 MTS we made a series of EGFP constructs containing regions of the N-terminus. EGFP fused to the first 162 or 80 amino acids of ADCK3 colocalised with MitoTracker^®^ Deep Red as determined by live cell fluorescence microscopy (S5A and S5B Fig). Interestingly, EGFP conjugated to the first 40 amino acids showed a more complex profile. Here, the majority of transfected cells displayed an EGFP signal that was split between the cytoplasm and mitochondria, indicating a problem with mitochondrial import of EGFP tagged protein (S5A and S5B Fig). A large population of cells also displayed diffuse cytoplasmic staining, indicative of a failure to import EGFP. These data suggested that the first 80 amino acids of ADCK3 were necessary and sufficient for the efficient import of EGFP into mitochondria. We extended this study by generating several truncated variants of ADCK3. Removing even the first 40 amino acids of the N-terminal MTS abrogated mitochondrial import of ADCK3 (S5C and S5D Fig), while deletion of amino acids 81–162 had little effect on mitochondrial import (S5C and S5D Fig). A similar result was also observed when amino acids 41–162 were deleted. We did observe, however, a reduced number of cells displaying an EGFP positive signal after transfection with the aa41-162Δ construct (data not shown), suggesting that loss of this region affected protein stability or expression. Thus, whilst the first 40 amino-acids of ADCK3 were important for mitochondrial import, amino-acids 41–162 appeared important for its stability/expression.

### adck3 mutant cells display signs of oxidative stress

**Fig 4:**
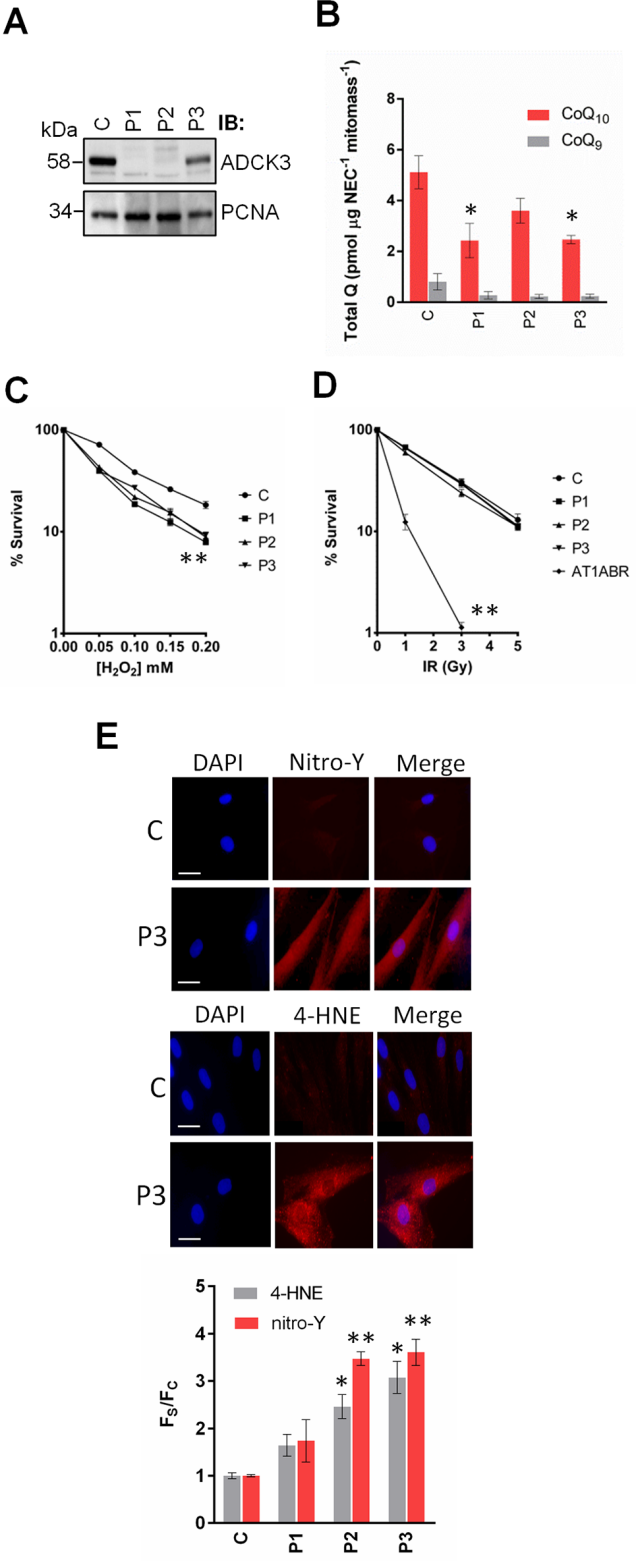
*adck3* mutant cells are sensitive to H_2_O_2_ and display an increase in oxidative stress. **(A).** Analysis of ADCK3 levels in control (C) and *adck3* mutant (P1, P2 and P3) mutant fibroblasts. WCEs (30 μg) were separated via SDS-PAGE and immunoblotting performed for ADCK3 and PCNA as a loading control. **(B).** Analysis of CoQ levels in *adck3* mutant cells. Total cellular levels of CoQ_10_ and CoQ_9_ were determined by RP-HPLC, normalised to non-esterified cholesterol and mitochondrial mass as described in Materials and Methods. **(C, D).** IR/H_2_O_2_ sensitivity of control and *adck3* mutant cell lines. Control and *adck3* mutant fibroblasts were exposed to various doses of H_2_O_2_ (C) or IR (D) and survival assessed via colony formation assays. AT1ABR is an Ataxia Telangiectasia (A-T) cell line used as a positive control for IR sensitivity. n = 3. **(E).** Analysis of 4-HNE Michael adducts and nitro-Y levels in *adck3* mutant cell lines. A representative example of the images obtained from this experiment are depicted. Fluorescence intensity values from individual cells were normalised to control values ± S. D. 50 cells analysed per experiment. n = 3. Statistical analysis was performed with Student’s t-test in all cases. * *p* < 0.05 and ** *p* < 0.005 (compared to control).

Having verified the localisation of ADCK3, we next sought to understand its cellular role. We investigated this through the use of primary cell lines that had previously been isolated from patients with ARCA-2 [24]. Using the anti-ADCK3 antibody we first analysed whether the nucleotide changes present in *adck3* mutant cell lines led to protein destabilisation. ADCK3 was undetectable in P1 cells, which contain a homozygous splice site mutation in the *ADCK3* gene, predicted to give rise to truncated protein (Table 1, Fig 4A). Interestingly, in P2 cells, which contain a homozygous triplet deletion in *ADCK3*, we observed a large reduction in ADCK3 protein levels. Finally, ADCK3 was present at reduced levels in P3 cells, consistent with the presence of a splice site acceptor mutation in one allele and a missense change in the other. Previous reports have shown that P2 and P3 but not P1 cells display a significant decrease in total CoQ_10_ content, the major form of coenzyme Q in human cells [24]. The side chain of CoQ is made from isoprenoid units that are 1) synthesised independently of Coq proteins and 2) are also the building blocks of cholesterol. Standardising CoQ data to cellular cholesterol content may therefore be more appropriate in terms of normalisation. Further normalisation of CoQ levels from this analysis to mitochondrial mass (see below), where the large majority of CoQ is synthesised in mammalian cells, led to the observation of significant changes in total CoQ_10_ levels (*i*.*e*. ubiquinone-10 plus ubiquinol-10) in P1 and P3 cells (Fig 4B). We also found that all of the mutant cell lines exhibited a significant increase in sensitivity to H_2_O_2_ (Fig 4C, *p* < 0.05) but not IR (Fig 4D). Immunofluorescence based experiments demonstrated that P2 and P3 cells also displayed a significant increase in the number of cells exhibiting an elevation of both protein nitrotyrosylation and 4-HNE Michael adduct formation (Fig 4E).

### adck3 mutant cells display changes in mitochondrial homeostasis and possible OXPHOS complex remodelling

**Fig 5:**
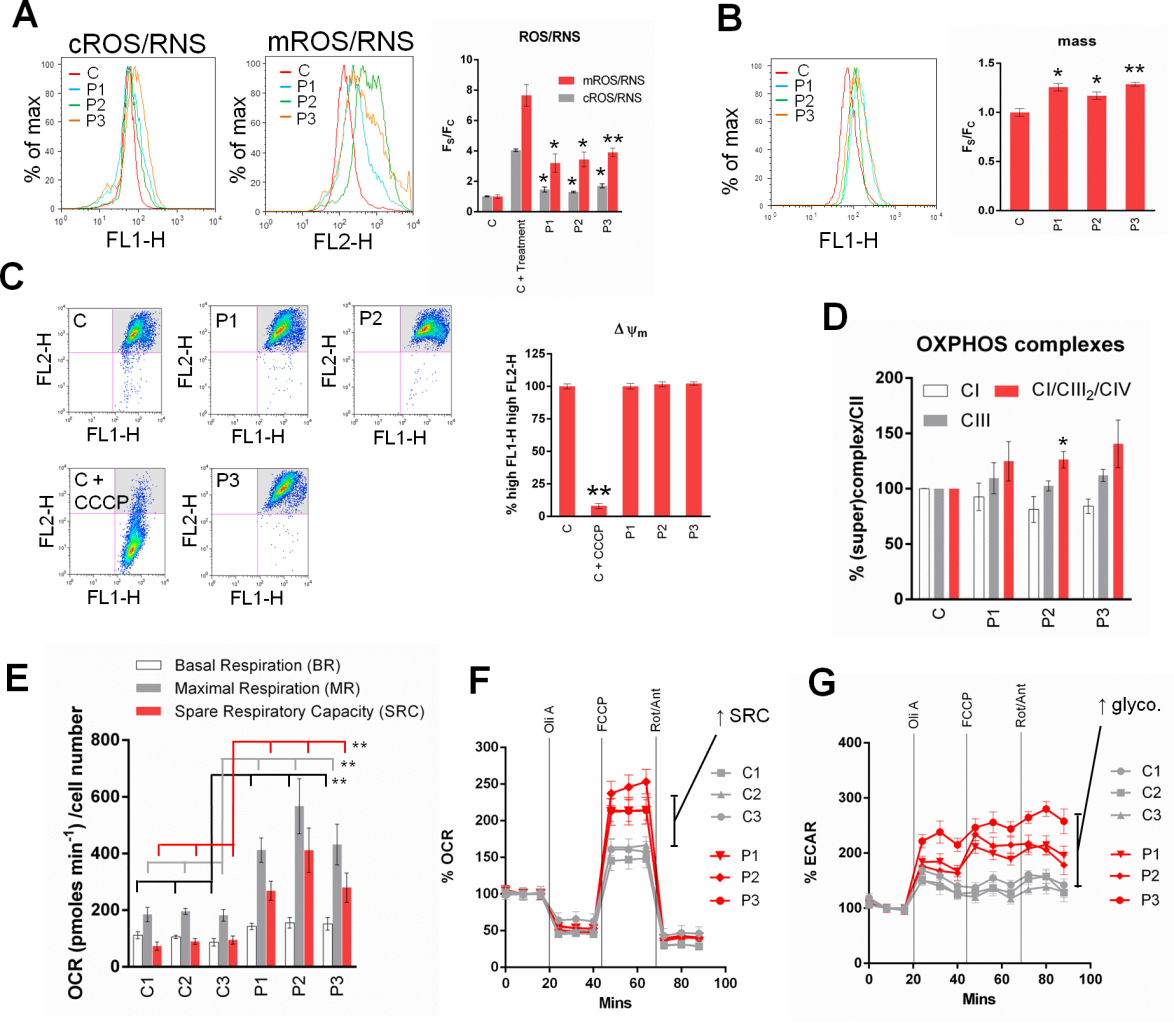
*adck3* mutant cells display changes in mitochondrial homeostasis and OXPHOS remodelling. **(A).** *adck3* mutant fibroblasts display a significant increase in cROS/RNS and mROS/RNS production. Analysis of cellular (cROS/RNS) and mitochondrial (mROS/RNS) ROS/RNS levels in control and *adck3* mutant fibroblasts under basal conditions. Cells stained with either 1 mM CM-H_2_-DCFDA (cROS/RNS) or 5 μM mitoSOX (mROS/RNS) and analysed via flow cytometry. C + treatment: 200 μM H_2_O_2_ (cROS/RNS) or 100 μM Antimycin A (mROS/RNS) for 1hr prior to staining with probes. Results are expressed as Mean Fluorescence Intensity divided by mitochondrial mass ((F_S_), also see B) and normalised to control values (F_C_). All data are from 3 independent experiments ± S. E. M. A representative histogram from flow cytometry is shown. **(B).** Mitochondrial mass is elevated in *adck3* mutant fibroblasts. Cells were stained with 1 μM NAO under basal conditions and analysed via flow cytometry. Results are expressed as Mean Fluorescence Intensity (F_S_) from 3 independent experiments (normalised to control values (F_C_)) ± S. E. M. A representative histogram from flow cytometry is shown. **(C).** ΔΨm is unaffected under basal conditions in *adck3* mutant cells. Control and *adck3* mutant fibroblasts were stained with JC-1 under basal conditions and analysed via flow cytometry. Results are expressed as % of cells with high FL1-H, high FL2-H signal (see grey shaded quadrant in representative dot plots) observed from 3 independent experiments ± S. E. M. **(D).** Analysis of OXPHOS (super)complex stability in control and *adck3* mutant cell lines. Isolated mitochondria solubilised in 1% Triton-X100 or 1% Digitonin prior to BN-PAGE and immunoblotting with anti-NDUFA9 (CI), anti-70 kDa (CII) and anti-Core I (CIII) (See S7 Fig). All data normalised to control values ± S. E. M. n = 3. **(E, F, G).** *adck3* mutant cells display an increase basal OCR and spare respiratory capacity. Oxygen consumption rate (OCR—F) and Extracellular Acidification Rate (ECAR—G) measurements in control and *adck3* mutant cells were acquired with a Seahorse Biosciences XF24 Analyzer. 20,000 cells per well. Oli (1 μM Oligomycin), FCCP (1 μM), Rot/Ant (1 μM Rotenone/Antimycin A). n = 3. Experimental values normalised to %OCR/ECAR in F, G. Raw data (OCR pmoles min^-1^ cell number^-1^) depicted in histogram presented in E. * *p* < 0.05; ** *p* < 0.005 (compared to control group) using Student’s t-test in (A, B, C) and Wilcoxon Mann Whitney test in (E).

Given the localisation of ADCK3 and its putative role in CoQ biosynthesis, we next investigated whether mitochondrial function was significantly affected in *adck3* mutant cells. As expected, *adck3* mutant fibroblasts displayed a significant increase in mROS/RNS production compared to control cells that was greatest in P3 cells (Fig 5A). There was also a small but significant increase in cROS/RNS production compared to control in the mutant cell lines. Increases in mROS/RNS production can parallel changes in mitochondrial mass. We tested whether this was true in *adck3* mutant fibroblasts using nonyl acridine orange. Here, we found a slight but significant increase in mitochondrial mass compared to control amongst the mutant cell lines (Fig 5B). However, mitochondrial morphology did not appear to be significantly affected in *adck3* mutant cells (S6 Fig). Moreover, there was no significant change in ΔΨ_m_ (Fig 5C) or OXPHOS complex stability (normalisation was performed to Complex II protein levels in all OXPHOS (super)complex measurements). There may have been a slight reduction in Complex I levels amongst the *adck3* mutant cell lines compared to control, although statistical analysis suggested that these changes were not significant (Fig 5D, S7A Fig). There did appear to be consistent elevation of OXPHOS supercomplex steady state levels (CI/CIII_2_/CIV) in the mutant cell lines compared to control (Fig 5D, S7B Fig), however, which was statistically significant in P2 cells. Furthermore, analysis of oxygen consumption rate in the presence of mitochondrial inhibitors suggested that all of the *adck3* cell lines had significantly increased basal and maximal respiration levels compared to controls, in addition to a significant increase in spare respiratory capacity (Fig 5E and 5F, *p* < 0.001 in all cases). This was accompanied by an increase in glycolytic flux after mitochondrial inhibition in all of the *adck3* mutant cell lines (Fig 5G). Together, these data suggested that loss of ADCK3 function leads to increases in both ROS/RNS production and mitochondrial mass with possible effects on OXPHOS (super)complex stability, oxygen consumption rates and glycolysis. Whilst it is known that deletion/mutation of Coq8 in *S*. *cerevisiae* leads to the appearance of unphosphorylated Coq3, Coq5 and Coq7 [30], we did not conduct any studies to see if this was the case in *adck3* mutant cells.

### adck3 mutant cells display an increase in lysosomal content

**Fig 6:**
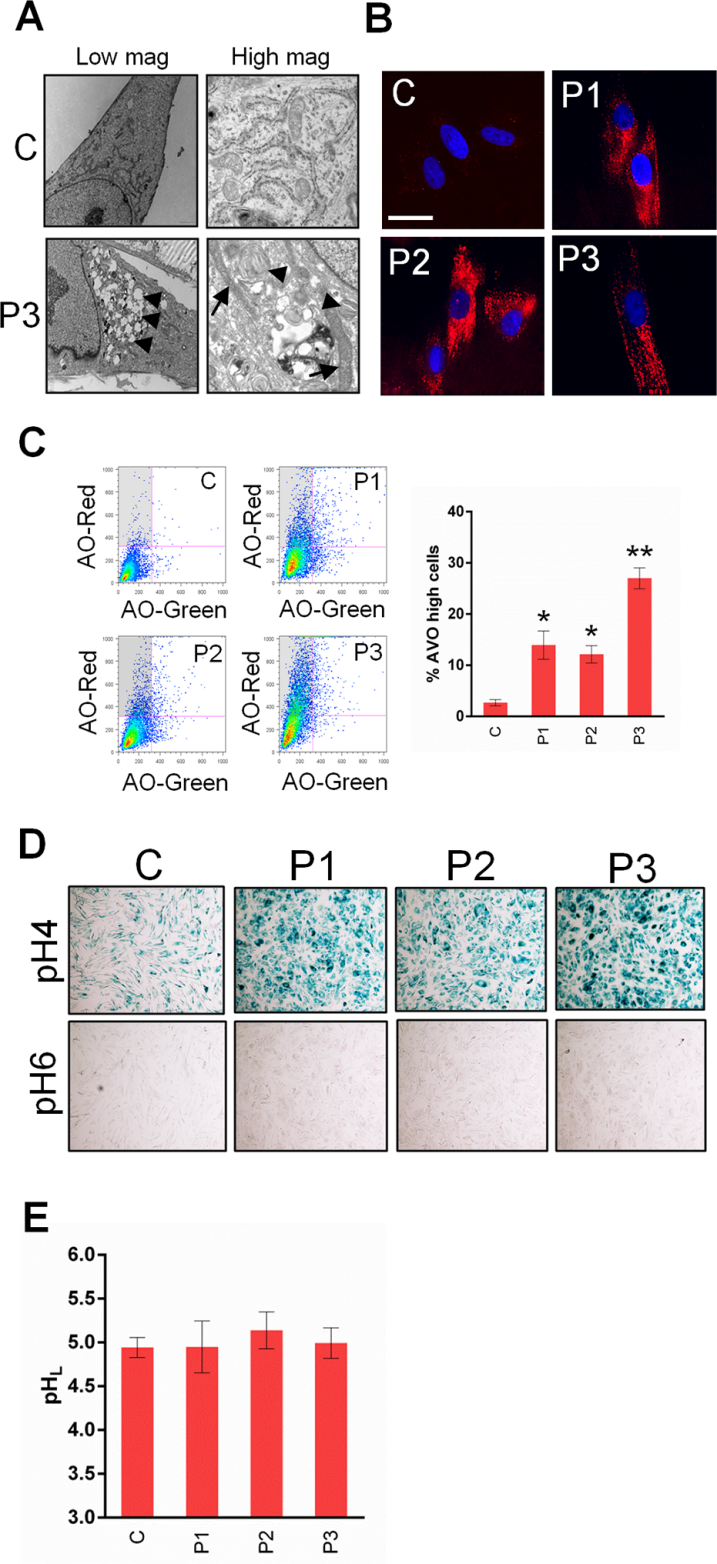
*adck3* mutant cells display an increase in lysosomal content but no effect on lysosomal pH under basal conditions. **(A).** Ultrastructural analysis of *adck3* mutant cells via TEM. An increase mitochondrial electron density and an increase in vesicular structures indicative of lysosomal expansion is observed in FD130 cells. In left panels, arrow heads indicate vesicular structures. In right panels, arrows indicate the position of electron dense mitochondria and arrow heads indicate electron dense lamellar bodies. **(B, C, D).** *adck3* mutant fibroblasts display a significant increase in lysosomal staining. Cells stained with lysotracker red (B) and acridine orange (C), or processed via β-galactosidase X-Gal staining were analysed via microscopy and flow cytometry. Results in (C) expressed as % acidic vesicle (AVO) positive cells (i.e. high AO-Red, low AO-Green–See grey shaded box in accompanying dotplots) normalised to control values ± S. E. M. n = 3. White bars: 30 μm. Images in (D) at 5x mag. **(E).** *adck3* cells show no change in lysosomal pH compared to control. Cells were stained with Lysosensor blue/yellow dextran for 24 h, chased for 6 h and analysed using a plate reader. n = 3. * *p* < 0.05 and *p* < 0.005 (compared to control) in all histograms using the Student’s t-test.

Although the mitochondrial network appeared to be normal in *adck3* mutant fibroblasts (S6 Fig) we were interested in whether there were any ultrastructural changes to mitochondria. EM depicted the presence of electron dense mitochondria in P3 but not control cells, corroborating our earlier data suggestive of mitochondrial dysfunction (Fig 6A, high magnification, arrows). Strikingly, the EM data also showed a large increase in the number of vacuoles present in P3 cells that were absent in control (Fig 6A, low magnification). Closer inspection also revealed the appearance of electron dense material and laminar bodies in P3 cells compared to control, indicative of an increase in the degradation of membranous material (Fig 6A, high magnification, arrow heads). Subsequent analysis of *adck3* cells using LysoTracker^®^ Red, acridine orange and β-galactosidase staining found that a significantly larger population of cells had elevated lysosomal staining compared to control (Fig 6B, 6C and 6D). We failed to observe any change in lysosomal pH, however, suggesting that the increase in lysosomal content was not due to changes in lysosomal acidification (Fig 6E).

**Fig 7:**
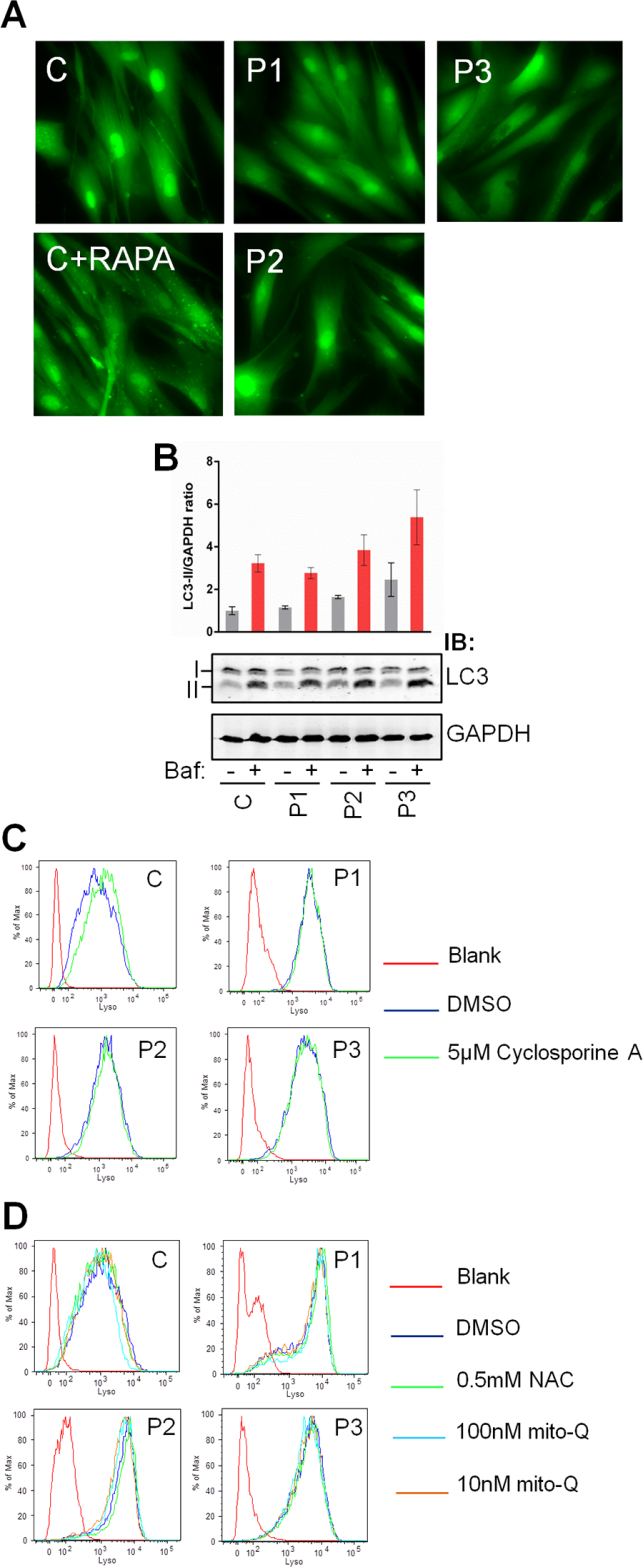
Analysis of autophagic flux and lysosomal staining in *adck3* mutant cells in response to antioxidant and Cyclosporine A treatment. **(A, B).** Analysis of autophagic flux in control and *adck3* mutant fibroblasts. GFP-LC3 transduced cells (A) were analysed via fluorescence microscopy for the appearance of GFP-LC3 puncta. RAPA– 1 mM Rapamycin treatment. WCEs (15 μg) from each cell line with or without bafilomycin A treatment (Baf) were also analysed via immunoblotting (B) with anti-LC3B and anti-GAPDH. Images were acquired and quantified using the Licor Odyssey IR based platform. I: LC3 Isoform I; II: LC3 Isoform II. Results displayed as LC3II/GAPDH ratio normalised to control values without bafilomycin treatment ± S. E. M. n = 3. = *p* ≤ 0.05 and = *p* ≤ 0.005 using Student’s t-test (compared to control) in all histograms. **(C, D).** Control and *adck3* mutant fibroblasts were incubated with DMSO, 0.5 mM N-acetyl cysteine (NAC), 10/100 nM mito-Q or 5 μM Cyclosporine A for 6 days in complete media prior to analysis using lysotracker red and flow cytometry. Media was changed every 2 days with the addition of fresh compound/s. n = 2. Statistical analysis by Student’s t-test showed no significant changes.

Given the rise in mROS/RNS production and appearance of membranous vesicles in P3 cells, we reasoned that the increase in lysosomal content observed in the *adck3* mutant cells may be due to an upregulation of mitochondrial turnover via mitophagy, as observed previously in other CoQ deficient fibroblasts [44]. We subsequently analysed the appearance of LC3-GFP puncta, a key marker of autophagosome formation, via fluorescence microscopy [45]. Although treatment with rapamycin (positive control) induced LC3-GFP puncta formation, there did not appear to be any significant increase in puncta formation in the *adck3* mutant cell lines under basal conditions when compared to control (Fig 7A). Furthermore, immunoblotting also failed to identify a significant increase in LC3-II levels under these conditions amongst the *adck3* mutant fibroblasts, again compared to control cells (Fig 7B). There was a slight increase in P3 cells, however, although this was not statistically significant. Prolonged treatment of cells with NAC, mitoQ or cyclosporine A also had little effect on lysosomal content (Fig 7C and 7D), suggesting that the upregulation of lysosomal staining was independent of mROS/RNS or changes in ΔΨm which are known to promote mitophagy [46, 47]. Together, these data suggest that the increase in lysosomal staining observed in *adck3* cells is independent of mitophagy induction.

## Discussion

*ADCK3* was originally identified as a p53 responsive gene and was later shown to be mutated in ARCA-2/SCAR9/COQ10D4 patients who display CoQ deficiency, cerebellar ataxia and exercise intolerance. In an attempt to gain further insight into ADCK3 function and how it might help protect against neurodegeneration, we sought to understand not only its precise cellular location, but its cellular role. Here, we show that ADCK3 contains an N-terminal MTS, localises to the matrix side of mitochondrial cristae and binds to components of the CoQ biosynthetic machinery *in vitro*. Finally, using cells lines isolated from ARCA-2 patients our data suggest that loss of ADCK3 function leads to sensitivity to hydrogen peroxide, high basal levels of oxidative stress, changes in mitochondrial metabolism and an increase in lysosomal content.

N-terminal localisation sequences targeting proteins to the mitochondrial compartment are generally alpha helical, amphipathic in nature and range from 10–80 amino acids in length [16]. Our data show that ADCK3 contains an unusually large MTS region with these characteristics (162 amino acids in length) and is cleaved at a possible MPP site (Fig 3). The nature of the cleavage site in ADCK3 is unusual in that it looks more similar to an R-10 motif (xRx↓(F/L/I)xx(S/T/G)xxxx↓) than an R-2/-3 site (xRx↓x(S/x)) [43]. Proteins with R-10 motifs tend to undergo two distinct cleavage events when imported into mitochondria—an initial MPP cleavage followed by a cleavage by mitochondrial intermediate peptidase (MIP), 8 amino acids downstream of the MPP cleavage site. N-terminal sequencing of exogenously expressed, mature protein isolated from WCEs indicated, however, that ADCK3 only underwent a single cleavage event after mitochondrial import at the putative MPP site. It is not immediately apparent why there is this difference although it is well known that the structure of the presequence, or even the mature portion of the protein, may hide the most important determinants for mitochondrial processing of any given precursor [43]. Indeed, this might also explain why the first 80 amino acids of the ADCK3 presequence were required for efficient import of EGFP into mitochondria but only the first 40 amino acids were required for ADCK3 import. As previously alluded to, the size of the MTS also appears to be larger than previously detailed for other mitochondrial proteins [48]. We initially thought that this region may be important in the targeting of ADCK3 to a specific sub-mitochondrial location. However, we found that ADCK3 in which amino acids 81–162 had been deleted still localised to mitochondrial cristae (data not shown). The large size of the MTS may therefore be required to regulate ADCK3 localisation, expression, or even stability, as suggested by our own truncation experiments. Our *in silico* analysis also shows that the ADCK3 MTS contains two possible CK II phosphorylation motifs, which may play a role in the import or stability of the protein (S8 Fig). Indeed, phosphorylation is known to regulate the activity/import of several mitochondrial proteins [49–52].

Our mitochondrial sub-fractionation experiments showed that after ADCK3 enters mitochondria it becomes peripherally associated with the IMM (Fig 1). This corroborates with recent data in budding yeast, which indicates that the ADCK3 orthologue, Coq8, binds in a non-integral manner to the matrix side of the IMM [29, 30]. Whether this is through direct interaction with the membrane or due to interactions with other proteins embedded in the IMM is unknown. Again, yeast based data has demonstrated that Coq8 interacts in a weak or transient manner with the CoQ biosynthetic complex [29]. Consistent with this, we were unable to detect ADCK3 in any high molecular weight complex under our BN-PAGE conditions. This conflicts with our own pull down data, however, which showed a relatively stable interaction between ADCK3 and its putative substrates (Coq3, Coq5, Coq7 and potentially Coq9) *in vitro*. One possible explanation for this discrepancy is that ADCK3 may promote the association of CoQ biosynthetic complex components to form the complex but is not part of the final complex *per se*. This corroborates with observations which suggest that Coq8 is detectable in a smaller size complex (consisting of Coq2, Coq5 and Coq10), which may be an intermediate of the *bone fide* CoQ biosynthetic complex in yeast [29]. Of course, the interactions we observe between ADCK3 and the CoQ complex components *in vitro* are more than certainly to be regulated differently *in vivo*.

Finally, we performed detailed phenotypic characterisation of cell lines isolated from ARCA-2 patients in an attempt to understand the cellular role of ADCK3. Mutant fibroblasts displayed sensitivity to oxidative-stress inducing agents, consistent with previous reports in fission yeast [53]. Our data also show that ADCK3 stability was differentially affected by mutation (Fig 4). Under basal conditions in DMEM media with glucose, loss/mutation of ADCK3 also significantly affected mitochondrial homeostasis, as observed by a significant upregulation of mROS/RNS production compared to control cells (Fig 5). ΔΨ_m_ was unaffected under these conditions, however, suggesting that loss of ADCK3 does not severely affect mitochondrial health in this glucose rich setting. This phenotype is distinct from that of fibroblasts isolated from patients with mutations in catalytic components of the CoQ biosynthetic machinery e.g. *COQ2*, which tend to show larger defects in CoQ levels/biosynthesis and mitochondrial membrane potential under basal conditions using similar cell culture media [44, 54]. Using these culture conditions we also observed consistent elevation of OXPHOS supercomplex stability amongst the *adck3* mutant fibroblasts, and a significantly large increase in basal and maximal respiration rates when compared to controls. These data also correlate with the increase in mROS/RNS production in these cells, suggesting that remodelling of OXPHOS supercomplexes might be occurring in the absence of ADCK3 function. Changes in supercomplex stability have recently been shown to modulate optimal ATP production and are dependent on substrate availability [55]. Perhaps loss of ADCK3 results in a compensatory change in respirasome formation, leading to an increase in flux through the electron transport chain that maintains ΔΨ_m_ in response to adverse conditions. Consistent with this notion is the fact that *adck3* mutant cells also displayed increased spare respiratory capacity in glucose containing DMEM after treatment with suboptimal concentrations of FCCP. These data conflict with other studies using the same *adck3* mutant fibroblasts, however, which suggest that mitochondrial ROS/RNS production and lipid peroxidation are not significantly affected by loss of ADCK3 function, even in galactose containing media in which mitochondrial dysfunction should be exacerbated [54]. Differences in culture conditions, media or assays used between this and the previous study may explain this discrepancy.

Interestingly, we found evidence for lysosomal accumulation in the *adck3* mutant cell lines, similar to what has previously been observed in a number of lysosome storage disorders (Fig 6). Our data also suggest that the increase in lysosomal staining observed was independent of major changes in autophagic flux or mitophagy. This is also different from cells isolated from patients with mutations in *COQ2*, where significant increases in LC3-II levels and cytochrome C colocalisation with LAMP1 were observed [44]. As stated above, loss of Coq2 function results in a much larger decrease in CoQ levels than loss of ADCK3. This affects mitochondrial homeostasis to a larger degree, resulting in loss of membrane potential, lysosomal accumulation and mitophagy induction. There are other forms of mitochondrial turnover, however, which can utilise lysosomes for the degradation of mitochondrial components without autophagosome involvement. The first of these utilises mitochondrial derived vesicles (MDV) to shuttle oxidatively damaged proteins from mitochondria to lysosomes [56, 57]. Treatment of *adck3* cells with antioxidants had no effect on lysosomal staining (Fig 7), however, suggesting that MDV formation might not be the cause of the increase in lysosomal numbers. An additional form of lysosome mediated mitochondrial turnover has recently been uncovered but again, this form occurs in response to the oxidative damage of mitochondrial proteins [58]. We also thought that loss of *adck3* might be affecting lysosomes to a greater extent than mitochondria in these cells, thus explaining the increase in lysosomal content. Indeed, there is a report which suggests that CoQ itself plays a role in regulating lysosomal function [59]. However, we saw no change in lysosomal pH in the absence of ADCK3, consistent with the mild defect in CoQ in these cells. Indeed, it is possible that the change in lysosomal content is CoQ independent and is due to loss of a non-canonical function of ADCK3 or compensatory response of cells. Interestingly, changes in lysosomal content have also been seen in cell lines isolated from patients with other autosomal recessive cerebellar ataxias, including ataxia telangiectasia[60], suggesting that lysosomal accumulation is a consistent feature of these syndromes. Whether the lysosomal compartment displays issues with degradative capacity or not in the absence of ADCK3 function also requires further investigation.

In summary, these data support the hypothesis that ADCK3 plays a role in CoQ biosynthesis and interacts with the core biosynthetic machinery. Our data also suggests that whilst loss of ADCK3 gives rise to lysosomal abnormalities, compensatory changes also occur in mitochondrial metabolism which improve respiratory capacity in response to stress. Together, these data shed light on ADCK3 function and highlight potential avenues to explore in determining the molecular basis of neurodegeneration in ARCA-2 patients.

## Supporting Information

S1 Fig Analysis of ADCK3 colocalisation with Mitotracker Deep Red staining over multiple z-axis positions. **(A).** Colocalisation of ADCK3-EGFP and Mitotracker® Deep Red. HeLa cells transiently transfected with ADCK3-EGFP (see S1 Table). Counterstaining for mitochondria was performed with Mitotracker® Deep Red (Mitotracker). Nuclei were stained with Hoechst 3342. White bars: 15 μm. 63x mag. Images acquired from multiple z-axis positions **(B).** Colocalisation of endogenous ADCK3 with Mitotracker Deep Red. Immunofluorescence based analysis of HeLa cells conducted with anti-ADCK3 and Alexa488-conjugated secondary antibodies (ADCK3) over multiple z-axis positions.
(TIF)

S2 Fig Analysis of ADCK3 Ab and ADCK3 expression in cell lines. **(A).** Knockdown of ADCK3 using siRNA. Single siRNA duplex (*ADCK3 #1*) was transfected into HeLa cells. WCEs generated at 48 h post transfection were subjected to immunoblot analysis with antibodies to ADCK3 and β actin. U: Untransfected samples. Arrow indicates possible ADCK3 protein band. * indicates possible non-specific band. **(B).** Analysis of ADCK3 expression in cell lines using the anti-ADCK3 antibody. **(C).** Anti-ADCK3 antibody immunoprecipitates ADCK3-FLAG. WCEs generated from HeLa cells transiently transfected with or without pcDNA3.1/Hygro(+)-*ADCK3*-FLAG (ADCK3-FLAG) were used for immunoprecipitation with either the anti-ADCK3 antibody (ADCK3) or a non-specific rabbit isotype control (IgG). Immunoblotting was performed following SDS-PAGE using anti-ADCK3 and anti-FLAG M2 antibodies.
(TIF)

S3 Fig Analysis of ADCK3 staining in mutant cell lines and siRNA treated HeLa cells. Analysis of endogenous ADCK3 staining (using anti-ADCK3 antibody) in *adck3* mutant cell lines. Arrow heads: perinuclear/’golgi-like’ staining.
(TIF)

S4 Fig *In vitro* import of ^35^S labelled ADCK3 at lower temperatures. *In vitro* import of S-methionine labelled ADCK3 into isolated mitochondria at lower temperatures i.e. 20°C and 4°C. Lysate lane shows separation of *in vitro* translated protein prior to incubation with mitochondria. Autorad image is displayed. p: precursor. m: mature protein. PK: Proteinase K. Note absence of import in 4°C experiment.
(TIF)

S5 Fig Analysis of the ADCK3 N-terminal MTS. **(A, B).** αα1–80 of ADCK3 are required for the efficient import of EGFP into mitochondria. Live cell imaging of HeLa cells transiently transfected with pEGFP-N3 based constructs containing αα1–162, αα1–80 or αα1–40 of ADCK3 (A). Counterstaining performed with Mitotracker Deep Red (Mitotracker) and Hoechst 3342 48 h post transfection prior to fluorescence microscopy. White bars: 15 μm. The proportion of cells which displayed a mitochondrial (Mito), cytoplasmic (Cyto) or cytoplasmic/mitochondrial (Cyto/Mito) EGFP signal was also determined (B). Data expressed as mean values normalised to control ± S. E. M. 200 cells scored in total from two independent experiments. **(C, D).** αα1–40 of ADCK3 are required for the efficient import of ADCK3 into mitochondria. Live cell imaging (C) and counterstaining performed as in A. Scoring of mitochondrial, cytoplasmic or split localisation of the ADCK3 variants (D, E) was performed as detailed in (B).
(TIF)

S6 Fig Mitochondrial morphology analysis. Mitochondrial morphology is unchanged in *adck3* fibroblasts. Cells were stained with mitotracker deep red and imaged via fluorescence microscopy. White bars: 30μm 63x mag. Representative images from 3 distinct experiments are shown.
(TIF)

S7 Fig Analysis of OXPHOS (super)complex stability. **(A, B).** Isolated mitochondria solubilised in 1% Triton-X100 (A) or 1% Digitonin (B) prior to BN-PAGE and immunoblotting with anti-NDUFA9 (CI), anti-70 kDa (CII) and anti-Core I (CIII).
(TIF)

S8 Fig *In silico* analysis of ADCK3 reveals the presence of sequence motifs related to several post-translational modifications. Results from PROSITE motif search (PredictProtein server) of ADCK3 (Isoform 1). N-Myristilation (N-MYR) can anchor proteins to membranes. Amidation is believed to promote structural flexability. Putative protein kinase C (PKC), tyrosine kinase (TYRK) and caesin kinase II (CKII) sites are also depicted. The MTS cleavage site is depicted by an arrow. Dashed green rectangle: Region conserved amongst ADCK family members and specifically related to CoQ biosynthesis. Dashed blue rectangle: Kinase-Like Domain (KLD). Dashed red rectangle: C-terminal region, conserved in the ADCK3/4 subgroup but divergent amongst typical protein kinases and other ADCK family members. Note the presence of possible CKII phosphorylation motifs in the MTS.
(TIF)

S9 Fig Full images of immunoblots detailed in Figs 1 and 2. Full images with molecular weight marker details for Fig 1Cii (A), Fig 1D (B), Fig 1F (C), Fig 1G (D) and Fig 2A (E) are depicted.
(TIF)

S10 Fig Full images of immunoblots detailed in Figs 3, 4 and 7. Full images with molecular weight marker deatils for Fig 3B (A), Fig 4A (B) and Fig 7B (C—Odyssey colour and grayscale projections are shown) are depicted.
(TIF)

S1 Table Plasmids used in this study. The plasmids used in this study together with details about their construction can be seen in the indicated table.
(DOCX)

S2 Table Primers used in this study. The primers used and the pertinent features of them are shown.
(DOCX)

## Abbreviations

ADCK: aarF domain containing kinase
ARCA-2: Autosomal Recessive Cerebellar Ataxia Type-2
CoQ: coenzyme Q
EGFP: enhanced green fluorescent protein
cROS/RNS: cellular reactive oxygen species/reactive nitrogen species
mROS/RNS: mitochondrial reactive oxygen species/reactive nitrogen species
4HNE: 4-hydroxyneonel
ΔΨ_m_: mitochondrial membrane potential
