# Correction: AarF Domain Containing Kinase 3 (ADCK3) Mutant Cells Display Signs of Oxidative Stress, Defects in Mitochondrial Homeostasis and Lysosomal Accumulation

**DOI:** 10.1371/journal.pone.0160162

**Published:** 2016-07-21

**Authors:** Jason K. Cullen, Norazian Abdul Murad, Abrey Yeo, Matthew McKenzie, Micheal Ward, Kok Leong Chong, Nicole L. Schieber, Robert G. Parton, Yi Chieh Lim, Ernst Wolvetang, Ghassan J. Maghzal, Roland Stocker, Martin F. Lavin

The following reference was incorrectly omitted: Stefely JA, Reidenbach AG, Ulbrich A, Oruganty K, Floyd BJ, et al. (2015) Mitochondrial ADCK3 Employs an Atypical Protein Kinase-like Fold to Enable Coenzyme Q Biosynthesis. Molecular Cell 57(1): 83–94, ISSN 1097-2765, http://dx.doi.org/10.1016/j.molcel.2014.11.002.
