# Supplementary figures and images for: AarF Domain Containing Kinase 3 (ADCK3) Mutant Cells Display Signs of Oxidative Stress, Defects in Mitochondrial Homeostasis and Lysosomal Accumulation
Source: PLoS One. 2016 Feb 11;11(2):e0148213. doi: 10.1371/journal.pone.0148213 (PMC4751082; doi:10.1371/journal.pone.0148213)

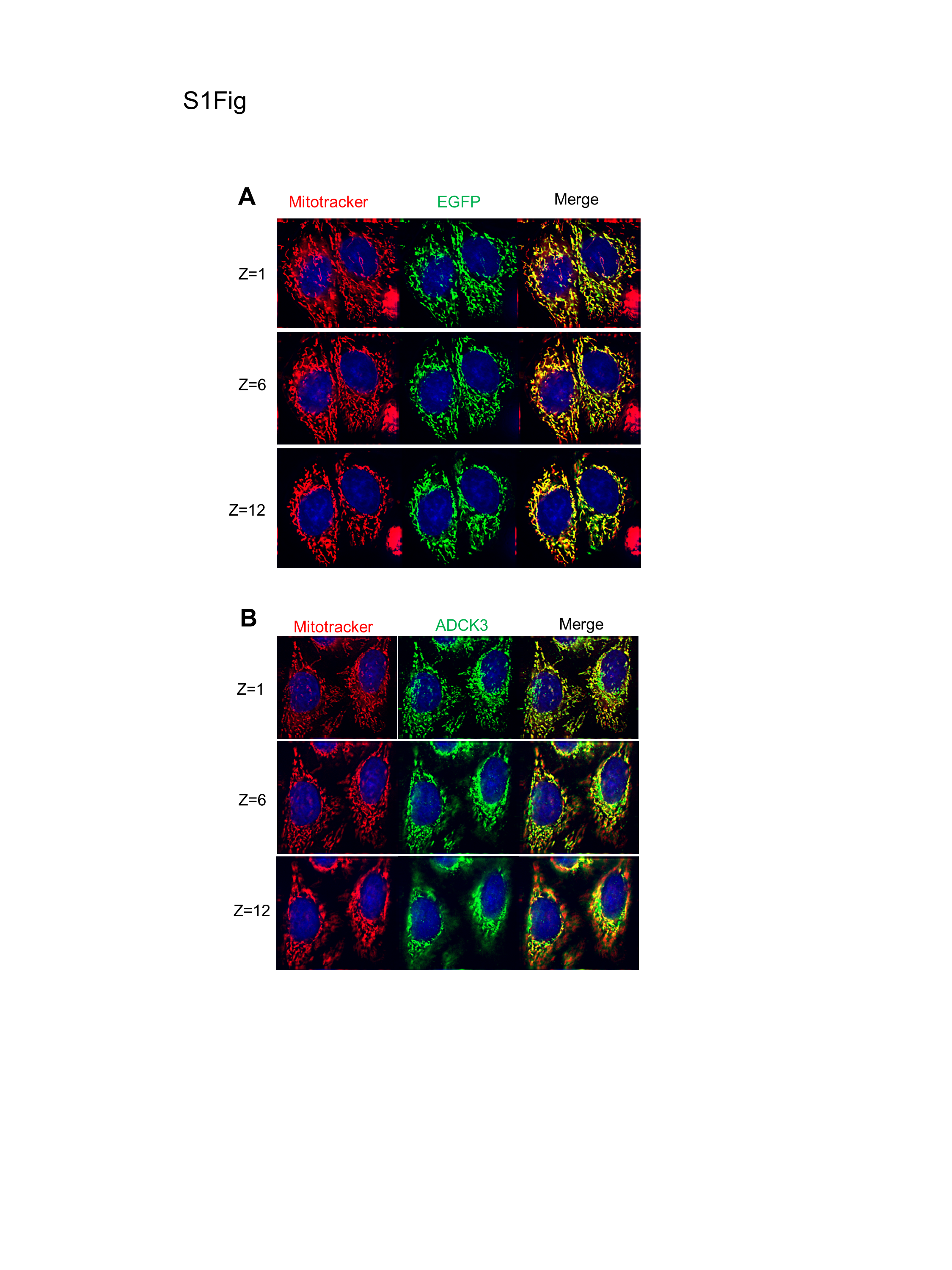

Supplement: S1 Fig — (A). Colocalisation of ADCK3-EGFP and Mitotracker® Deep Red. HeLa cells transiently transfected with ADCK3-EGFP (see S1 Table). Counterstaining for mitochondria was performed with Mitotracker® Deep Red (Mitotracker). Nuclei were stained with Hoechst 3342. White bars: 15 μm. 63x mag. Images acquired from multiple z-axis positions (B). Colocalisation of endogenous ADCK3 with Mitotracker Deep Red. Immunofluorescence based analysis of HeLa cells conducted with anti-ADCK3 and Alexa488-conjugated secondary antibodies (ADCK3) over multiple z-axis positions. (TIF) [file pone.0148213.s001.tif]

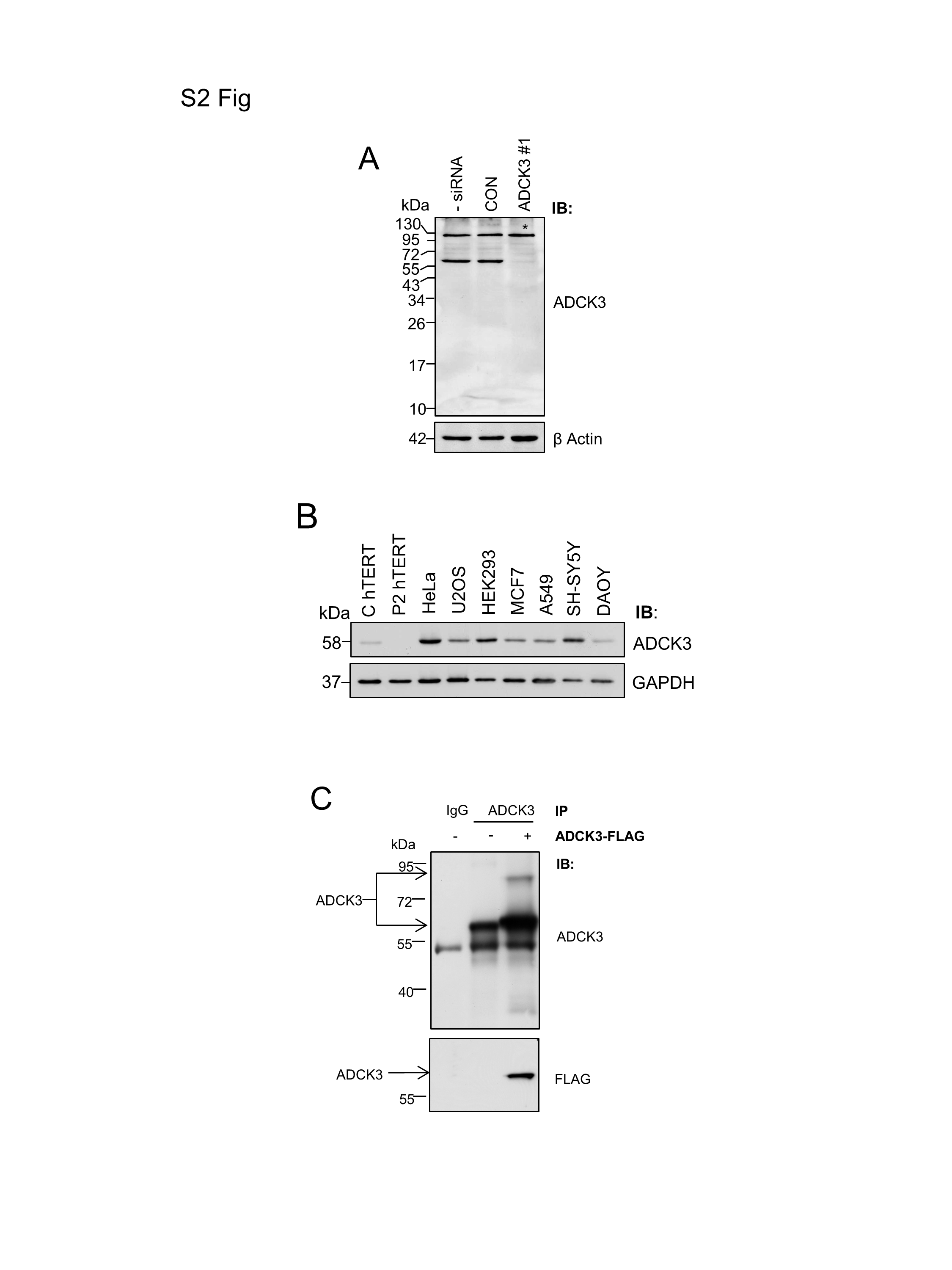

Supplement: S2 Fig — (A). Knockdown of ADCK3 using siRNA. Single siRNA duplex (ADCK3 #1) was transfected into HeLa cells. WCEs generated at 48 h post transfection were subjected to immunoblot analysis with antibodies to ADCK3 and β actin. U: Untransfected samples. Arrow indicates possible ADCK3 protein band. * indicates possible non-specific band. (B). Analysis of ADCK3 expression in cell lines using the anti-ADCK3 antibody. (C). Anti-ADCK3 antibody immunoprecipitates ADCK3-FLAG. WCEs generated from HeLa cells transiently transfected with or without pcDNA3.1/Hygro(+)-ADCK3-FLAG (ADCK3-FLAG) were used for immunoprecipitation with either the anti-ADCK3 antibody (ADCK3) or a non-specific rabbit isotype control (IgG). Immunoblotting was performed following SDS-PAGE using anti-ADCK3 and anti-FLAG M2 antibodies. (TIF) [file pone.0148213.s002.tif]

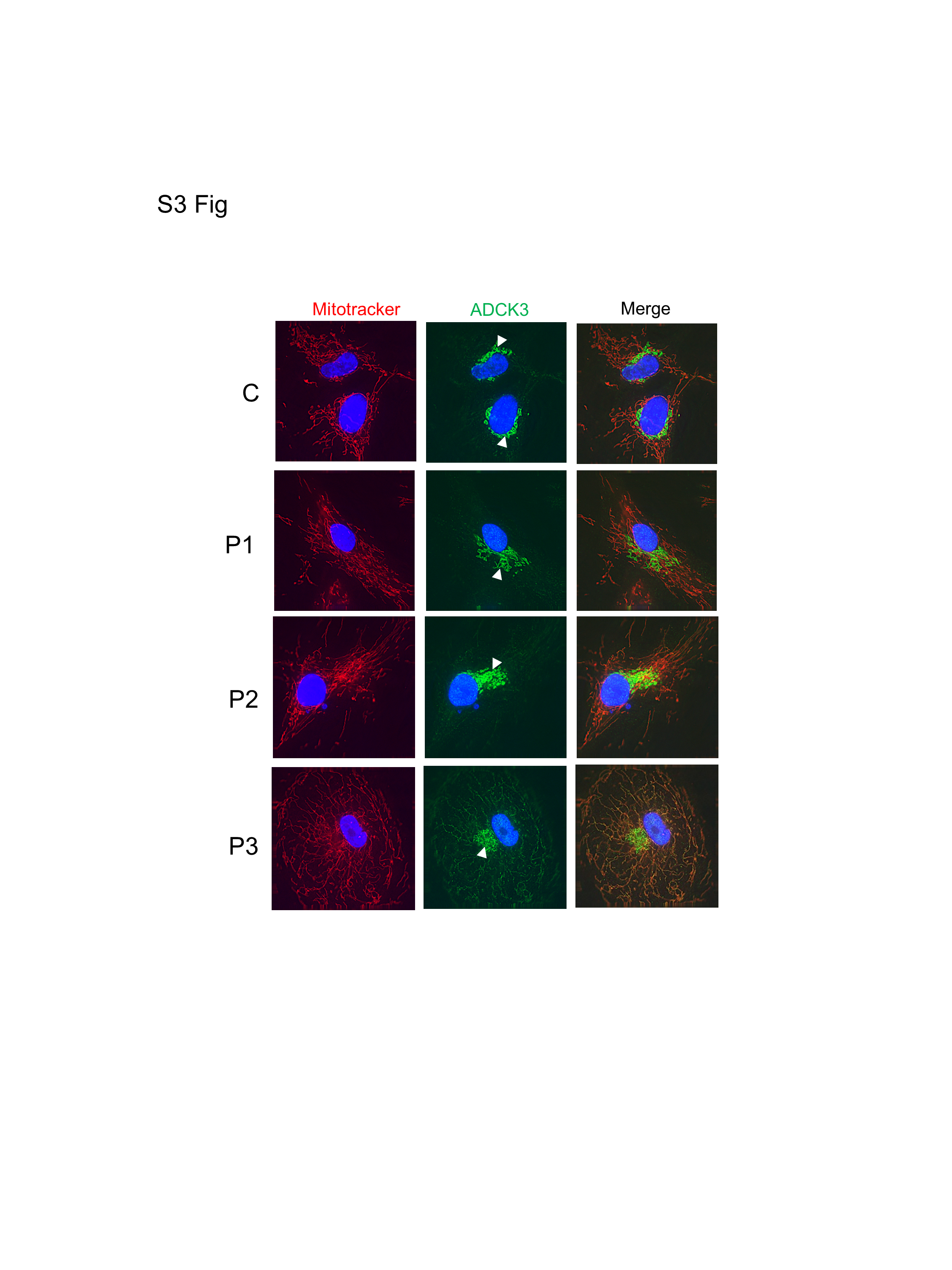

Supplement: S3 Fig — Analysis of endogenous ADCK3 staining (using anti-ADCK3 antibody) in adck3 mutant cell lines. Arrow heads: perinuclear/’golgi-like’ staining. (TIF) [file pone.0148213.s003.tif]

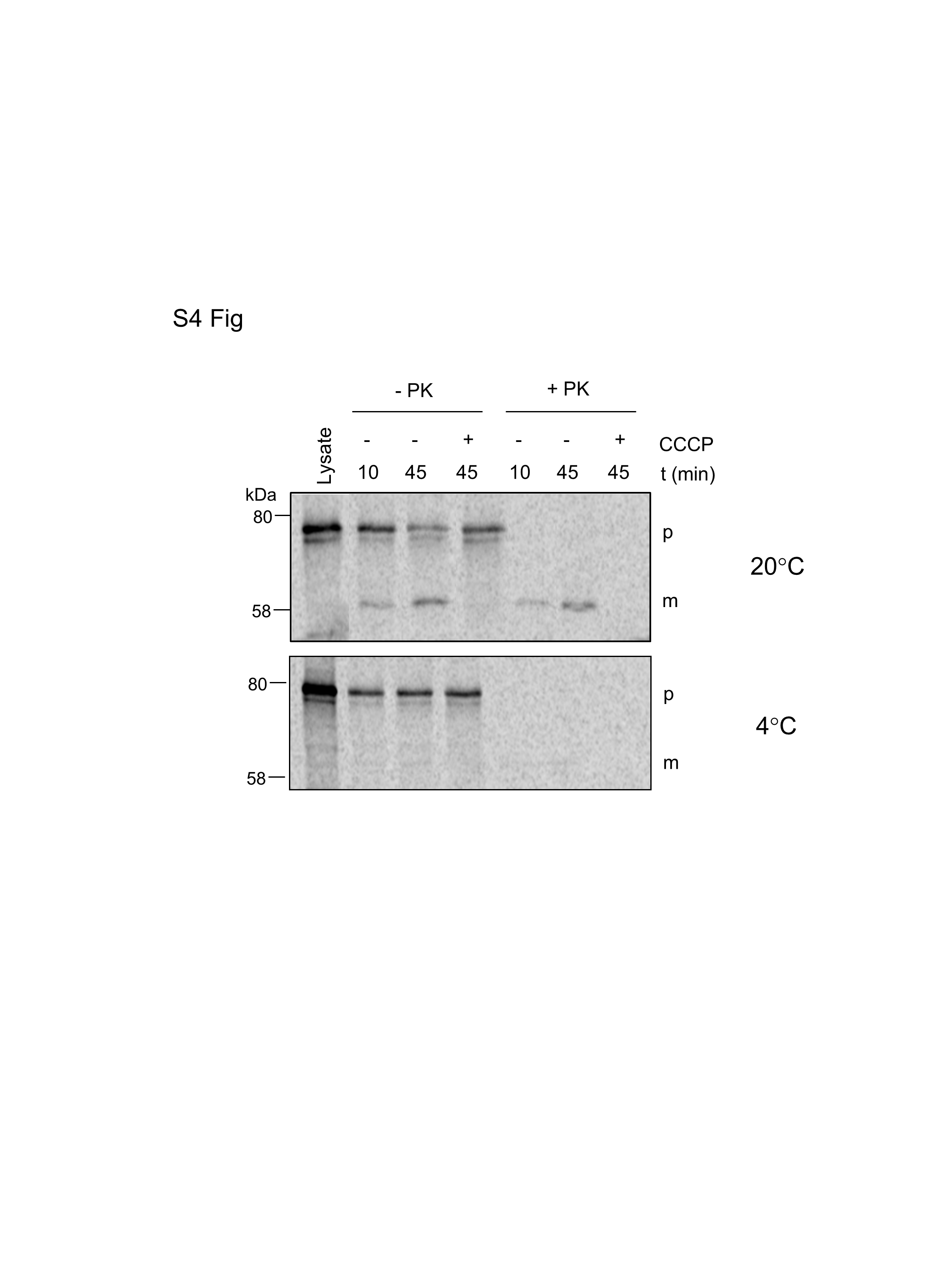

Supplement: S4 Fig — In vitro import of S-methionine labelled ADCK3 into isolated mitochondria at lower temperatures i.e. 20°C and 4°C. Lysate lane shows separation of in vitro translated protein prior to incubation with mitochondria. Autorad image is displayed. p: precursor. m: mature protein. PK: Proteinase K. Note absence of import in 4°C experiment. (TIF) [file pone.0148213.s004.tif]

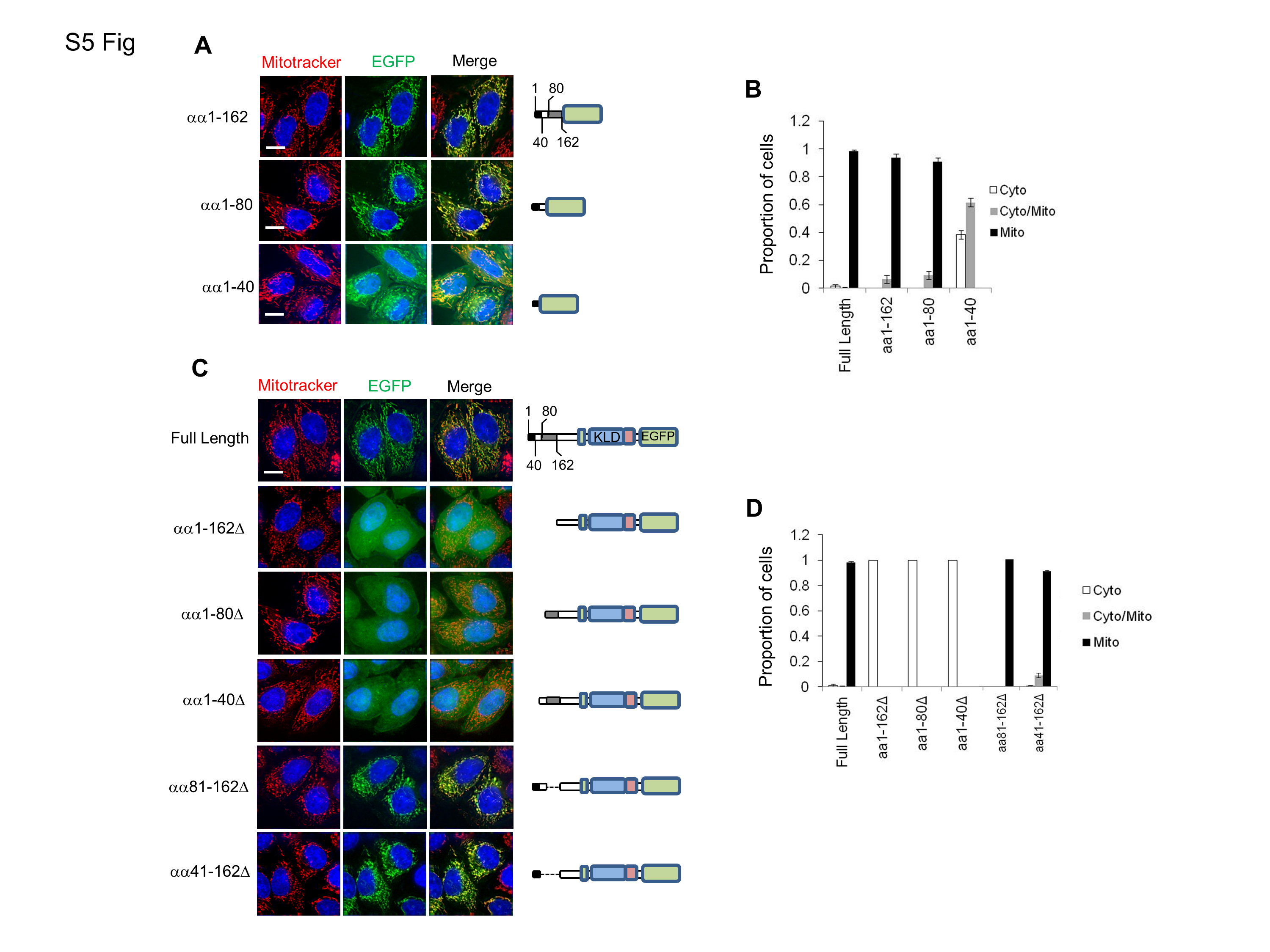

Supplement: S5 Fig — (A, B). αα1–80 of ADCK3 are required for the efficient import of EGFP into mitochondria. Live cell imaging of HeLa cells transiently transfected with pEGFP-N3 based constructs containing αα1–162, αα1–80 or αα1–40 of ADCK3 (A). Counterstaining performed with Mitotracker Deep Red (Mitotracker) and Hoechst 3342 48 h post transfection prior to fluorescence microscopy. White bars: 15 μm. The proportion of cells which displayed a mitochondrial (Mito), cytoplasmic (Cyto) or cytoplasmic/mitochondrial (Cyto/Mito) EGFP signal was also determined (B). Data expressed as mean values normalised to control ± S. E. M. 200 cells scored in total from two independent experiments. (C, D). αα1–40 of ADCK3 are required for the efficient import of ADCK3 into mitochondria. Live cell imaging (C) and counterstaining performed as in A. Scoring of mitochondrial, cytoplasmic or split localisation of the ADCK3 variants (D, E) was performed as detailed in (B). (TIF) [file pone.0148213.s005.tif]

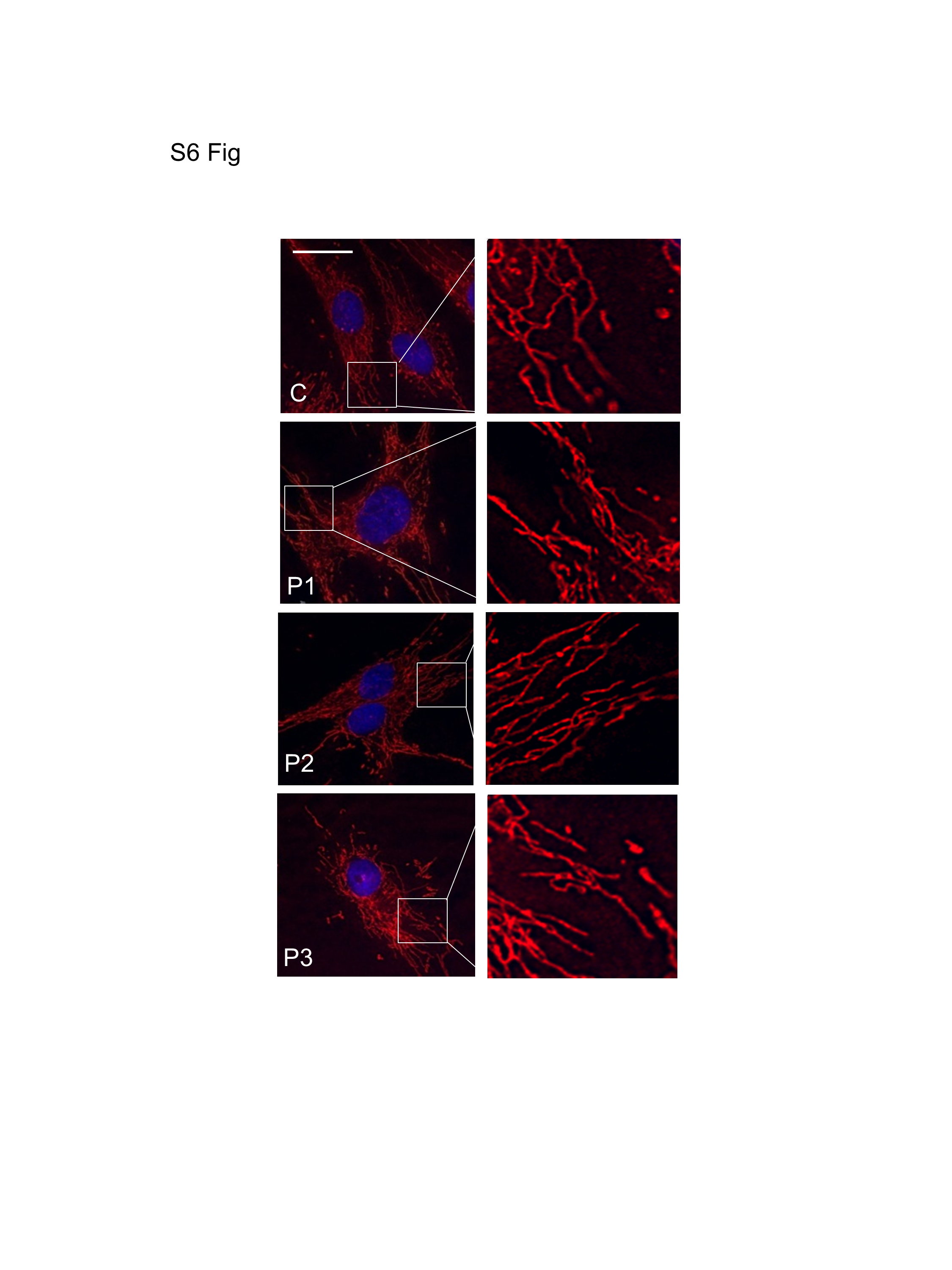

Supplement: S6 Fig — Mitochondrial morphology is unchanged in adck3 fibroblasts. Cells were stained with mitotracker deep red and imaged via fluorescence microscopy. White bars: 30μm 63x mag. Representative images from 3 distinct experiments are shown. (TIF) [file pone.0148213.s006.tif]

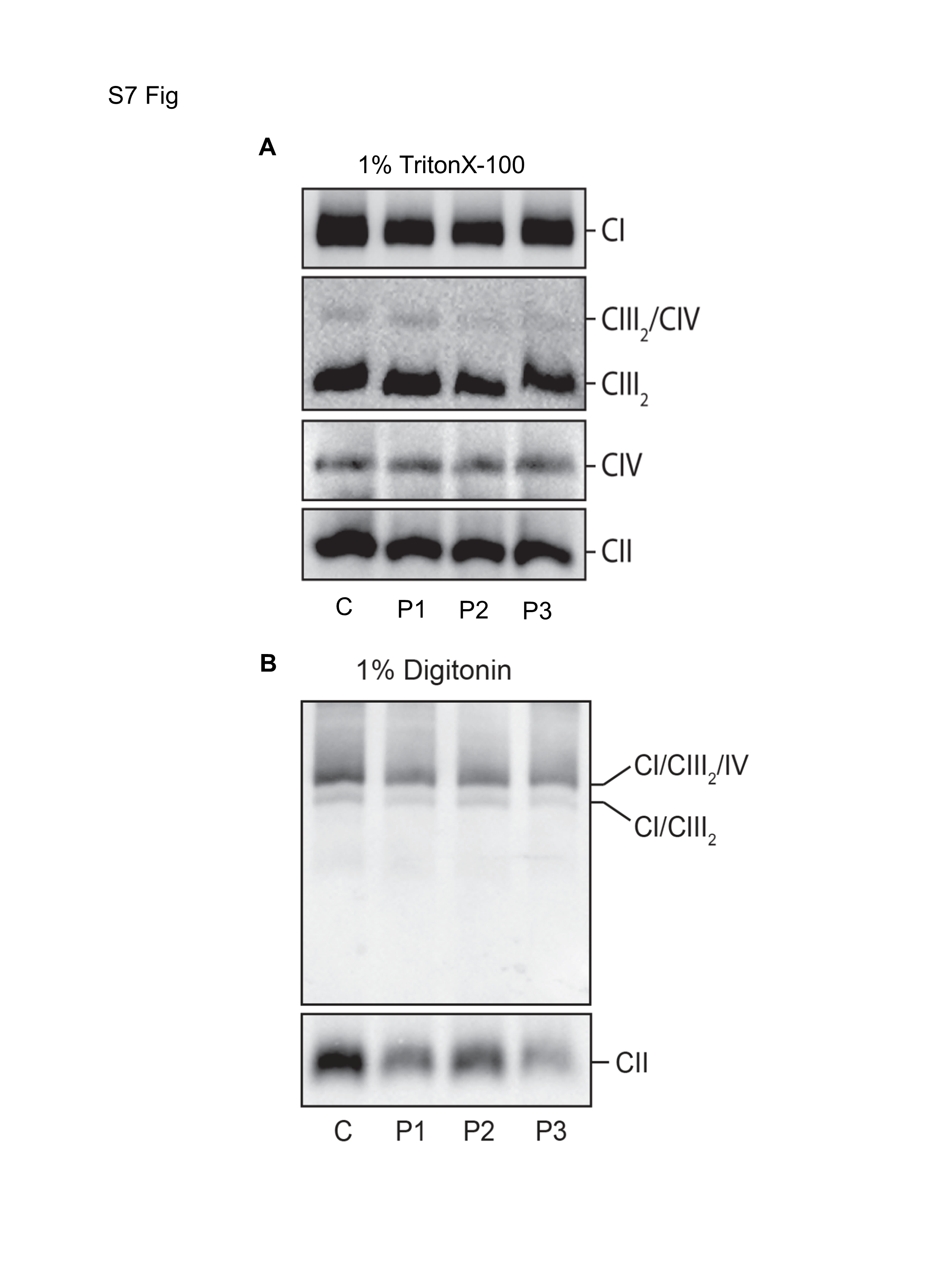

Supplement: S7 Fig — (A, B). Isolated mitochondria solubilised in 1% Triton-X100 (A) or 1% Digitonin (B) prior to BN-PAGE and immunoblotting with anti-NDUFA9 (CI), anti-70 kDa (CII) and anti-Core I (CIII). (TIF) [file pone.0148213.s007.tif]

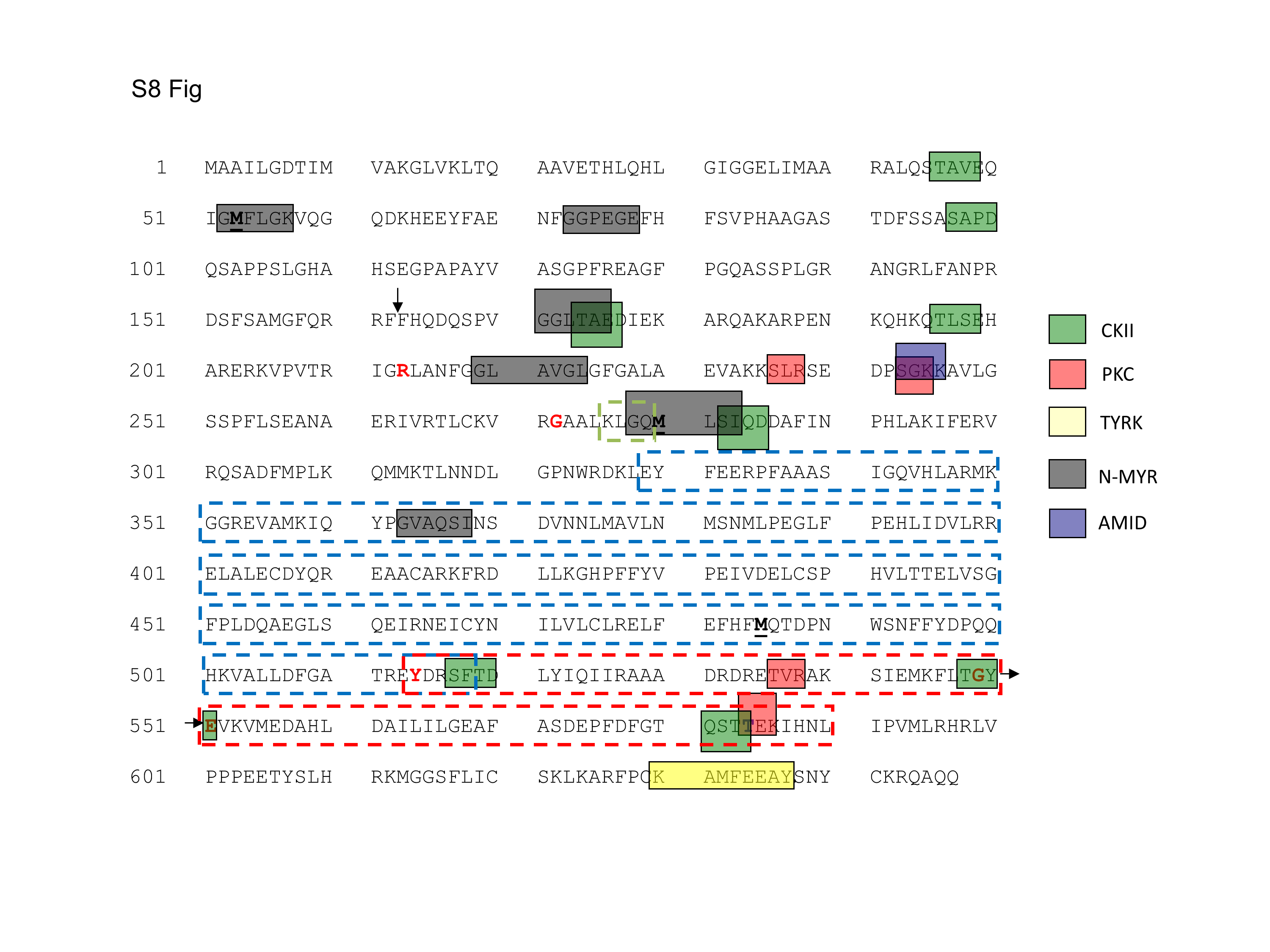

Supplement: S8 Fig — Results from PROSITE motif search (PredictProtein server) of ADCK3 (Isoform 1). N-Myristilation (N-MYR) can anchor proteins to membranes. Amidation is believed to promote structural flexability. Putative protein kinase C (PKC), tyrosine kinase (TYRK) and caesin kinase II (CKII) sites are also depicted. The MTS cleavage site is depicted by an arrow. Dashed green rectangle: Region conserved amongst ADCK family members and specifically related to CoQ biosynthesis. Dashed blue rectangle: Kinase-Like Domain (KLD). Dashed red rectangle: C-terminal region, conserved in the ADCK3/4 subgroup but divergent amongst typical protein kinases and other ADCK family members. Note the presence of possible CKII phosphorylation motifs in the MTS. (TIF) [file pone.0148213.s008.tif]

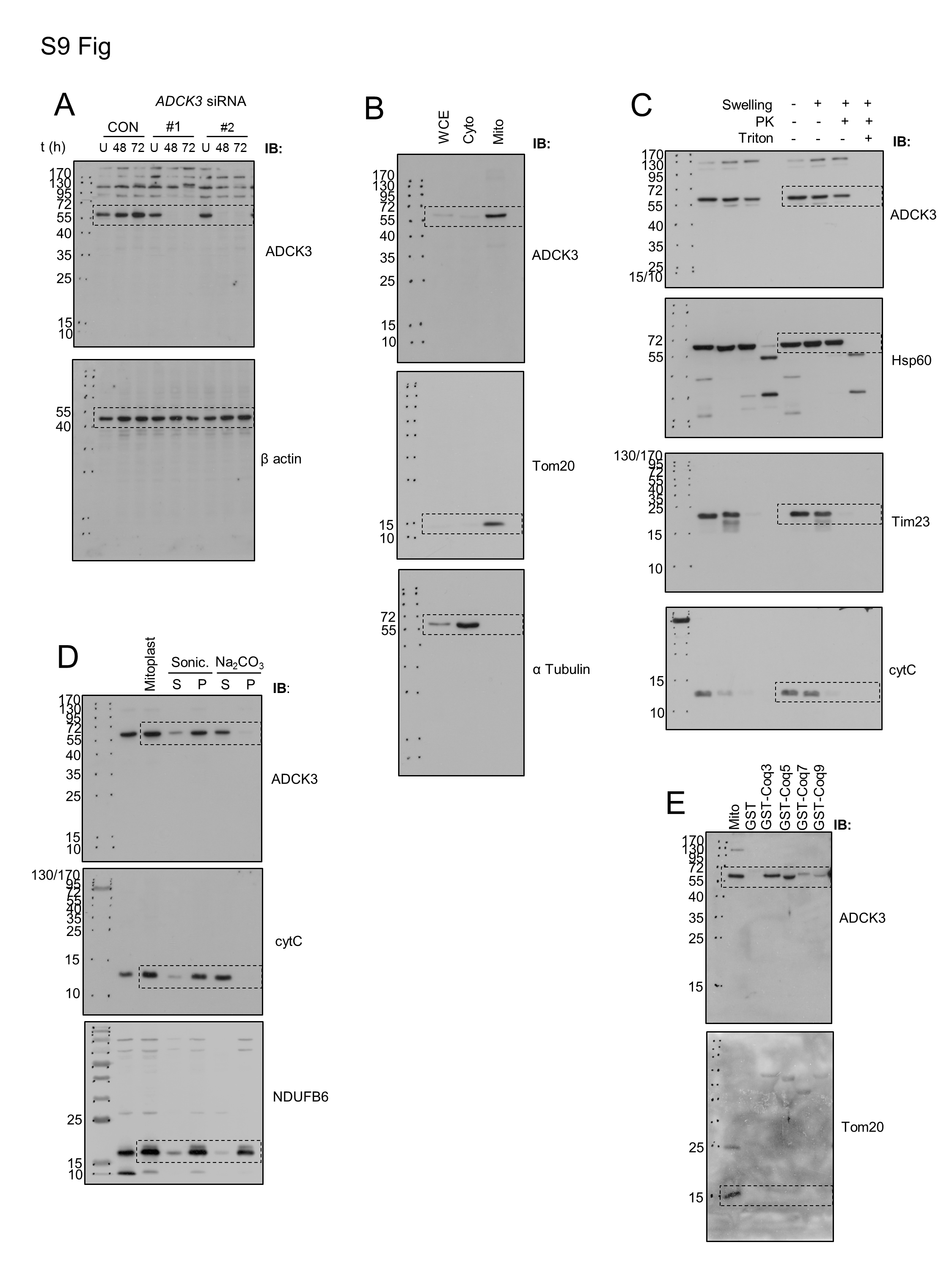

Supplement: S9 Fig — Full images with molecular weight marker details for Fig 1Cii (A), Fig 1D (B), Fig 1F (C), Fig 1G (D) and Fig 2A (E) are depicted. (TIF) [file pone.0148213.s009.tif]

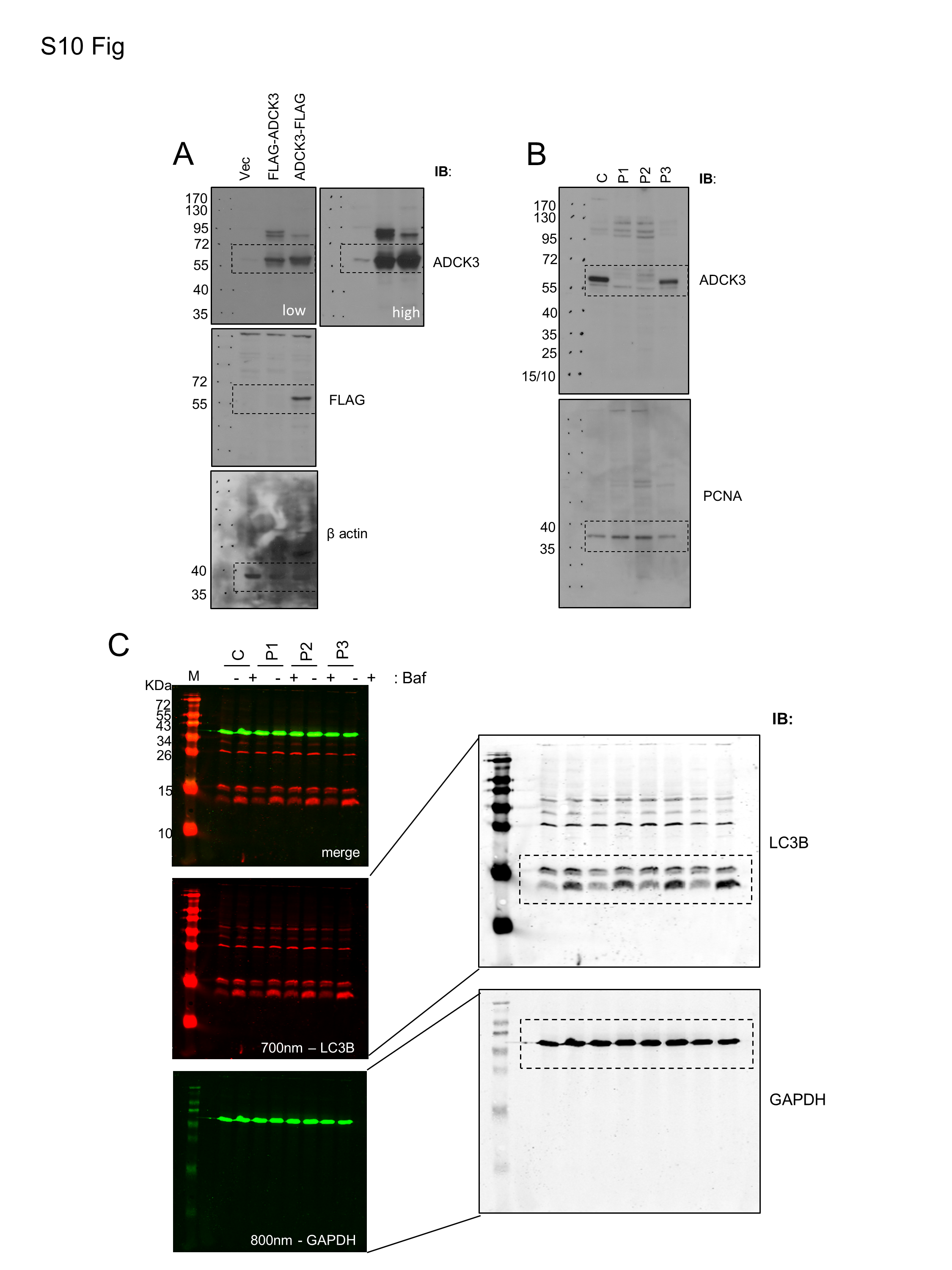

Supplement: S10 Fig — Full images with molecular weight marker deatils for Fig 3B (A), Fig 4A (B) and Fig 7B (C—Odyssey colour and grayscale projections are shown) are depicted. (TIF) [file pone.0148213.s010.tif]
