# Supplementary material for: AarF Domain Containing Kinase 3 (ADCK3) Mutant Cells Display Signs of Oxidative Stress, Defects in Mitochondrial Homeostasis and Lysosomal Accumulation
Source: PLoS One. 2016 Feb 11;11(2):e0148213. doi: 10.1371/journal.pone.0148213 (PMC4751082; doi:10.1371/journal.pone.0148213)
Supplement: S1 Table — The plasmids used in this study together with details about their construction can be seen in the indicated table. (DOCX) [file pone.0148213.s011.docx]

**S1 Table. Plasmids used in this study.**

| **Plasmid** | **Features** |
| --- | --- |
| pcDNA3.1Hygro(+)-FLAG-*ADCK3* | cDNA subcloned from pDNR-Dual plasmid containing *ADCK3* ORF (Clone ID: HsCD00022398 - plasmID). N-terminal FLAG tag on ADCK3. |
| pcDNA3.1Hygro(+)-*ADCK3-*FLAG | C-terminal FLAG tag on ADCK3. cDNA subcloned from pDNR-Dual as above. |
| pGEM4Z-*ADCK3* | *In vitro* transcription/translation of ADCK3 for use in mitochondrial import assays. cDNA subcloned from pDNR-Dual as above. |
| pEGFP-*ADCK3* | C terminal EGFP tag on ADCK3. cDNA subcloned from pDNR-Dual as above. |
| pEGFP-*ADCK3-­*163-647Δ | C-terminal EGFP tag on aa1-162 of ADCK3. cDNA subcloned from pDNR-Dual as above. |
| pEGFP-*ADCK3-­*81-647Δ | C-terminal EGFP tag on aa1-80 of ADCK3. cDNA subcloned from pDNR-Dual as above. |
| pEGFP-*ADCK3-­*41-647Δ | C-terminal EGFP tag on aa1-40 of ADCK3. cDNA subcloned from pDNR-Dual as above. |
| pEGFP-*ADCK3-­*1-162Δ | Deletion of aa1-162 of ADCK3. cDNA subcloned from pDNR-Dual as above. |
| pEGFP-*ADCK3-­*1-80Δ | Deletion of aa1-80 of ADCK3. cDNA subcloned from pDNR-Dual as above. |
| pEGFP-*ADCK3-­*1-40Δ | Deletion of aa1-40 of ADCK3. cDNA subcloned from pDNR-Dual as above. |
| pEGFP-*ADCK3-­*81-162Δ | Deletion of aa81-162 of ADCK3. cDNA corresponding to aa1-80 of ADCK3 was subcloned from pDNR-Dual as above and inserted into pEGFP-*ADCK3-­*1-162Δ construct. |
| pEGFP-*ADCK3-­*41-162Δ | Deletion of aa41-162 of ADCK3. cDNA corresponding to aa1-40 of ADCK3 was subcloned from pDNR-Dual as above and inserted into pEGFP-*ADCK3-­*1-162Δ construct. |
| pGEX-6P-1-*COQ3* | cDNA subcloned from pENTR223 plasmid containing *COQ3* ORF (Clone ID: [HsCD00288664](http://plasmid.med.harvard.edu/PLASMID/GetCloneDetail.do?cloneid=288664&species=) - plasmID). |
| pGEX-6P-1-*COQ5* | cDNA subcloned from pDNR-LIB plasmid containing *COQ5* ORF (Clone ID: [HsCD00339987](http://plasmid.med.harvard.edu/PLASMID/GetCloneDetail.do?cloneid=288664&species=) - plasmID). |
| pGEX-6P-1-*COQ7* | cDNA subcloned from pDONR201 plasmid containing *COQ7* ORF (Clone ID: [HsCD00082097](http://plasmid.med.harvard.edu/PLASMID/GetCloneDetail.do?cloneid=288664&species=) - plasmID). |
| pGEX-6P-1-*COQ9* | cDNA subcloned from pENTR223 plasmid containing *COQ9* ORF (Clone ID: [HsCD00288635](http://plasmid.med.harvard.edu/PLASMID/GetCloneDetail.do?cloneid=288664&species=) - plasmID). |
