# Supplementary material for: AarF Domain Containing Kinase 3 (ADCK3) Mutant Cells Display Signs of Oxidative Stress, Defects in Mitochondrial Homeostasis and Lysosomal Accumulation
Source: PLoS One. 2016 Feb 11;11(2):e0148213. doi: 10.1371/journal.pone.0148213 (PMC4751082; doi:10.1371/journal.pone.0148213)
Supplement: S2 Table — The primers used and the pertinent features of them are shown. (DOCX) [file pone.0148213.s012.docx]

**S2 Table. Primers used in this study.**

| **Primer** | **Sequence 5'-3'** | **Features** |
| --- | --- | --- |
| ADCK3.F8.EcoRI | GGAATTCATGGCTGCCATATTGGGAGACAC | ATG is present. 5' EcoRI restriction site. |
| ADCK3FLAGstop_BamHI_R | CGGGATCCCTATTTATCATCATCATCTTTATAATCCTGCTGGGCCTGCCTCTTGCAGTAGTTGCT | 3' BamHI site.  FLAG tag. Touchdown amplification with ADCK3.F8.EcoRI. Reverse |
| ATGFLAGADCK3_HindIII_F | CCCAAGCTTATGGATTATAAAGATGATGA | 5' HindIII site. |
| ADCK3.R2 | CGGGATCCCTACTGCTGGGCCTGCCTCTTGC | 3' BamHI restriction site. |
| ADCK3.F1 | CGGGATCCATGGCTGCCATATTGGGAGACAC | 5' BamHI restriction site. |
| ADCK3.F7 | GGGGTACCATGGCTGCCATATTGGGAGACAC | KpnI restriction site |
| ADCK3.R1.1-162 | CGGGATCCGGACCTTCGCTAAAGCC | BamHI restriction site |
| ADCK3.R2.1-80 | GGGGATCCGTGGAACTCCCCTTCTGGGC | BamHI restriction site |
| ADCK3.R1.1-40 | CGGGATCCGGCCGCCATGATCAGCTCCC | BamHI restriction site |
| ADCK3.F1.162-647 | GGGGTACCATGTTCCACCAGGACCAATCC | KpnI restriction site |
| ADCK3.F1.81-647 | GGGGTACCATGTTCTCAGTCCCGCATGCAGC | KpnI restriction site |
| ADCK3.F1.41-647 | GGGGTACCATGAGGGCCCTGCAGTCCACG | KpnI restriction site |
| COQ3.BamHI.F3 | CGGGATCCTGGAGTGGCCGTAAGCTGGG | 5' BamHI restriction site. |
| COQ3.EcoRI.R3 | GGAATTCTTATTTCTTCAGCTTTTCATGCACAGC | 3' EcoRI restriction site. |
| COQ5.EcoRI.F1 | GGAATTCGCGGCCCCCGGGAGCTGTGCC | 5' EcoRI restriction site. |
| COQ5.SalI.R1 | GGGTCGACTTAAAGTTTGAAGCCAGAATGAATGG | 3' SalI restriction site. |
| COQ7.BamHI.F3 | CGGGATCCAGTTGCGCCGGGGCGGCGGCGGC | 5' BamHI restriction site. |
| COQ7.EcoRI.R3 | GGAATTCTTATAATCTTTCTGATAAATATATCGC | 3' EcoRI restriction site. |
| COQ9.BamHI.F3 | CGGGATCCGCGGCGGCGGCGGTATCTGG | 5' BamHI restriction site. |
| COQ9.EcoRI.R3 | GGAATTCTTAGCACCGACGCTGGTTTAGACCT | 3' EcoRI restriction site. |
